# Four-Coordinate Fe N_2_ and Imido Complexes Supported by a Hemilabile NNC Heteroscorpionate Ligand

**DOI:** 10.1021/acs.inorgchem.2c01656

**Published:** 2022-07-27

**Authors:** Alex McSkimming, Niklas B. Thompson

**Affiliations:** † Department of Chemistry, Tulane University, New Orleans, Louisiana 70118, United States; ‡ Chemical Sciences and Engineering Division, Argonne National Laboratory, Lemont, Illinois 60439, United States

## Abstract

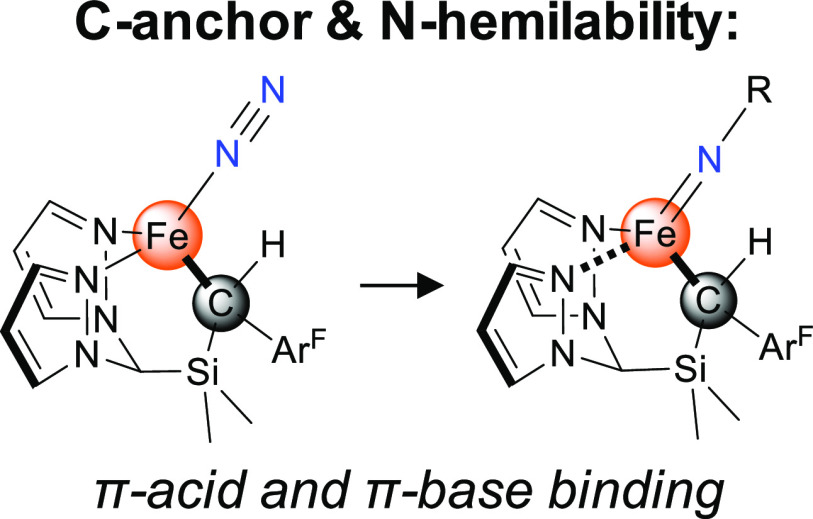

Inspired by mechanistic proposals for N_2_ reduction at the nitrogenase FeMo cofactor, we report herein a new, strongly σ-donating heteroscorpionate ligand featuring two weak-field pyrazoles and an alkyl donor. This ligand supports four-coordinate Fe(I)-N_2_, Fe(II)-Cl, and Fe(III)-imido complexes, which we have characterized using a variety of spectroscopic and computational methods. Structural and quantum mechanical analysis reveal the nature of the Fe–C bonds to be essentially invariant between the complexes, with conversion between the (formally) low-valent Fe-N_2_ and high-valent Fe-imido complexes mediated by pyrazole hemilability. This presents a useful strategy for substrate reduction at such low-coordinate centers and suggests a mechanism by which FeMoco might accommodate the binding of both π-acidic and π-basic nitrogenous substrates.

## Abstract 2

We report a new, strongly σ-donating NNC heteroscorpionate ligand and its Fe(I)-N_2_, Fe(II)-Cl and Fe(III)-imido complexes. Conversion between the low-valent Fe-N_2_ and high-valent Fe-imido complexes is mediated by pyrazole hemilability, presenting a useful strategy for substrate reduction at such low-coordinate centers.

## Introduction

**Figure 1:**
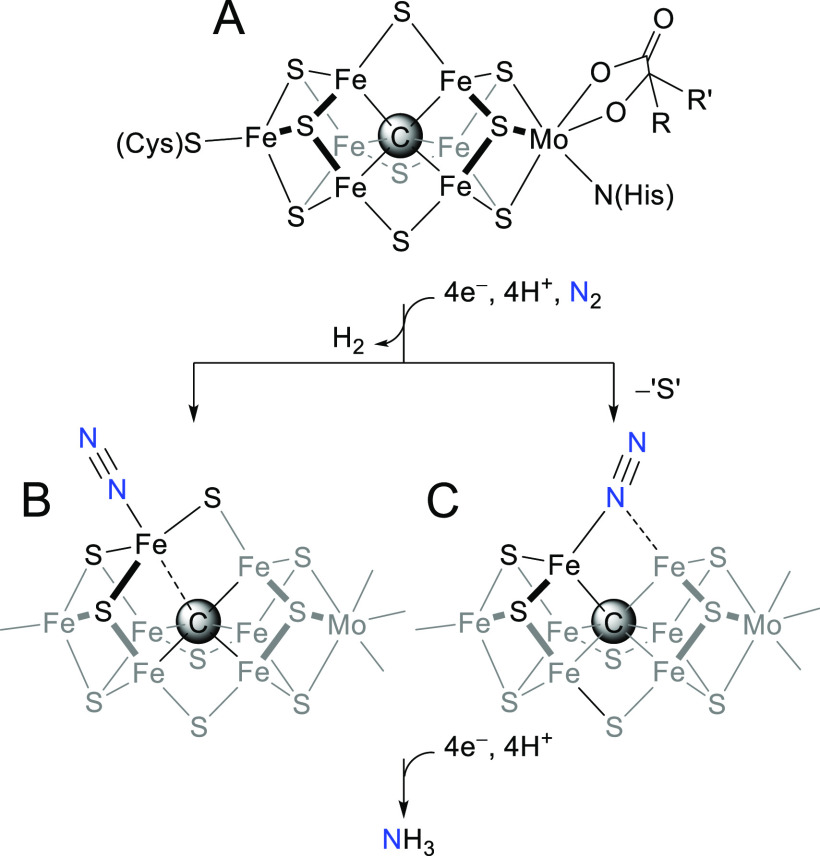
(A) Resting state of the FeMoco active site of Mo-dependent nitrogenases. (B, C) Truncated hypothetical, N_2_-bound structures for the “E4” FeMoco intermediate. The dashed lines indicate uncertain or elongated bonds. Protonation/oxidation states of the sulfides and Fe centers are not shown.

The reduction of atmospheric N_2_ into bioavailable NH_3_ by nitrogen-fixing microorganisms is an essential biogeochemical process.^1^ This reaction is catalyzed by nitrogenase enzymes that use a complex metallocluster, most commonly the iron–molybdenum cofactor, or “FeMoco” (Figure 1A), to overcome the barrier associated with breaking the exceedingly strong N_2_ triple bond.^2^ Despite recent advances, a full mechanistic description for FeMoco has not been widely accepted.^3^ In addition, competent synthetic N_2_ reduction catalysts remain elusive, the development of which draws synergistically from biochemistry and inorganic coordination chemistry.^4^

An unusual structural feature of FeMoco is the “interstitial” light atom—now known to be carbon—at the center of the cluster (Figure 1A).^5,6^ As this carbide ligates the Fe atom(s) implicated as the site(s) of substrate binding and reduction,^7^ it is expected to play a critical, if currently obscured, role in catalysis. Often speculated is that the Fe–C bonds are hemilabile, thus allowing the substrate-bound Fe site to accommodate the diverse nuclear and electronic structures necessary to stabilize both π-acidic N_2_ and π-basic nitrogen hydrides (N_*x*_H_*y*_) during catalysis (Figure 1B).^8−12^ Recently, however, structural^13,14^ and EPR^15^ studies have revealed that carbon monoxide binds FeMoco *via* the displacement of a “belt” sulfide, which bridges two carbide-bound Fe centers. Plausibly, N_2_ and its reduction products could bind in much the same fashion (Figure 1C), although any such intermediates defy unequivocal structural characterization.^16−20^ These results suggest an alternative mechanism in which the interstitial carbide serves to “anchor” the substrate-bound Fe—which is expected to sample multiple coordination geometries and/or numbers—against dissociation.^15,21,22^

We have been interested in preparing synthetic, mononuclear metal complexes inspired by the latter hypothesis (*i.e.*, Figure 1C), for applications in the multi-electron reduction of unsaturated substrates. A common strategy for such chemistry is the use of “hard–soft” multi-dentate ligands that incorporate both a π-acceptor—such as a phosphine—to stabilize low-valent intermediates and a hard X-type donor—such as an amide—to stabilize high-valent intermediates.^23^ In contrast, we wished to prepare four-coordinate metal complexes supported by a rigid, facially coordinating tridentate ligand devoid of π-acceptors, thus ensuring the strong donicity required to activate N_2_, CO, *etc*. Akin to the coordination environment of an Fe site in FeMoco, such a ligand would feature a strongly σ-donating C-group and two weak-field donors. We envisaged that the covalent M–C bond would avert complete ligand dissociation, with the more labile interactions between the metal center and the weak-field donors fluctuating to accommodate changes in metal oxidation state and bonding at the unique ligand site.^24^

**Scheme 1:**
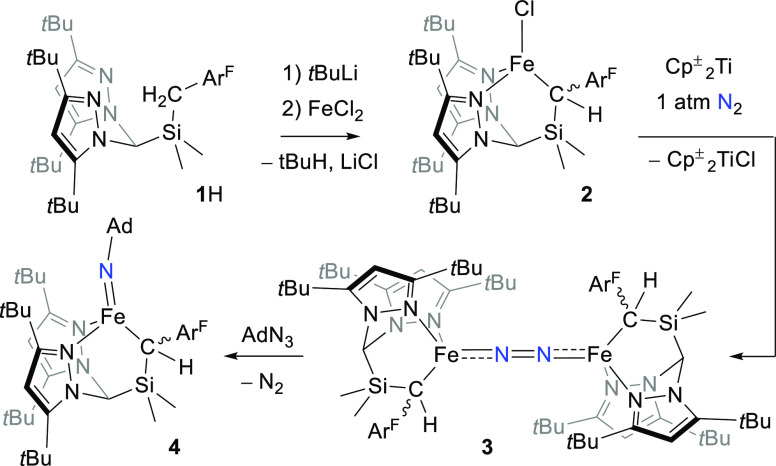
Synthesis of Metal Complexes Ar^F^ = 3,5-(CF_3_)_2_C_6_H_3_; Cp^±^ = C_5_Me_4_(SiMe_3_); Ad = 1-adamantyl.

Accordingly, we report herein a new scorpionate ligand platform, **1** (Scheme 1), which contains two weak-field pyrazoles and an alkyl donor. This ligand supports four-coordinate Fe centers spanning three formal oxidation states, including N_2_ and imido complexes. Structural analysis combined with quantum chemical calculations reveal that static Fe–C bonds, combined with a more dynamic Fe–pyrazole interaction, do indeed accommodate changes in the electronic structure of Fe induced by the N-terminal ligand.

## Experimental Section

### General Methods

All manipulations involving metal complexes were carried out in an N_2_ atmosphere glovebox. Glassware were oven-dried for at least several hours at 160 °C prior to use.

### Materials

All solvents except *n*-pentane were distilled from purple Na/benzophenone prior to use. All solvents were stored over activated 3 Å molecular sieves for at least 24 h prior to use. All reagents were purchased from commercial suppliers and used without further purification unless otherwise noted. HC(^*t*Bu^pz)_2_SiMe_2_Cl,^25^ (C_5_Me_4_(SiMe_3_))_2_Ti,^26^ and AdN_3_^27^ were prepared according to literature procedures.

### Spectroscopy and Spectrometry

NMR spectra were recorded on Bruker 300 or 400 MHz spectrometers. ^1^H and ^13^C chemical shifts are reported in ppm relative to tetramethylsilane using residual solvent as an internal standard. ^19^F chemical shifts are reported in ppm relative to 5% v/v internal PhF.^28^ Solution-phase effective magnetic moments were determined by the method described by Evans^29^ and are corrected for diamagnetic contributions (as are SQUID magnetometry data).^30^ Mass spectrometry data were collected on a Bruker MicroTOF II with an ESI source. FTIR spectra were recorded on solid samples using a Bruker Alpha II FTIR spectrometer operating at 4 cm^–1^ resolution. Elemental analyses were performed by Midwest Microlab. EPR spectra were recorded on a Bruker EMX spectrometer. Simulations were performed using EasySpin^31^ (5.2.33) in MATLAB (R2021b). DC magnetic susceptibility data for a microcrystalline sample of **4** were collected on an MPMS SQUID magnetometer in the range of 5–300 K with a 10,000 Oe applied field. The sample was prepared by compressing 12.2 mg of **4** between wads of quartz wool in a length of quartz tubing, which was then flame-sealed under vacuum. Simulations were performed using PHI^32^ (3.1.5).

### X-ray Crystallography

Low-temperature diffraction data were collected on a Bruker-AXS X8 Kappa Duo diffractometer coupled to an APEX2 CCD detector. The data collections were executed with Mo K_α_ radiation (λ = 0.71073 Å) from a *I*μ*S* microsource performing ϕ-and ω-scans. Absorption and other corrections were applied using the program SADABS.^33,34^ The structures were solved by dual-space methods using SHELXT^35^ and refined against *F*^2^ on all data by full-matrix least squares with SHELXL-2017^36^ following established refinement strategies.^37^ All non-hydrogen atoms were refined anisotropically, and all hydrogen atoms were included into the model at geometrically calculated positions and refined using a riding model.

### Synthesis of **1**H

We have found that for the scale described below, the initiation of the Grignard reaction is only mildly exothermic, and so, the following manipulations were performed in a glovebox. To a stirred suspension of Mg^0^ (∼0.80 g, ∼33 mmol) in Et_2_O (20 mL) was added roughly one-fifth of a solution of (3,5-(CF_3_)_2_C_6_H_3_)CH_2_Cl (4.44 g, 16.9 mmol) in Et_2_O (10 mL). The mixture was stirred until formation of the Grignard reagent became apparent, as indicated by a slight yellowing of the solution. At this stage, the remainder of the (3,5-(CF_3_)_2_C_6_H_3_)CH_2_Cl solution was added sufficiently slowly *via* pipette such that boiling of the Et_2_O remained well-controlled. After completion of the addition, the mixture was stirred until bubbling of the Et_2_O had ceased, at which point stirring was continued another 15 min. The solution of the Grignard reagent was then pipetted into a stirred solution of HC(^*t*Bu^pz)_2_SiMe_2_Cl (5.25 g, 11.3 mmol) in Et_2_O (10 mL) (no particular care is required for this addition). After several minutes, Mg^2+^ salts began to precipitate. Stirring was continued for 2 h, at which point the reaction mixture was removed from the glovebox and quenched carefully by the slow addition of water. The organic phase was separated and dried over sodium sulfate, and the solvent removed under reduced pressure. The crude yellow oil was dissolved in hexanes (∼10 mL) and passed through a short pad of silica (∼2 × 3 cm), eluting with hexanes until the product had fully eluted. Thoroughly removing all volatiles under reduced pressure gave the product as a thick, almost completely colorless oil. The product was ∼98% pure by NMR spectroscopy and sufficiently pure for further reactions. Yield: 6.56 g (89%). ^1^H NMR (300 MHz, C_6_D_6_): δ 7.60 (s, 1H, *p*-Ar^F^*H*), 7.47 (s, 2H, 2 × *o*-Ar^F^*H*), 6.93 (s, 1H, 2 × N_pz_–C*H*), 6.00 (s, 2H, 2 × pz*H*), 2.29 (s, 2H, C*H*_2_–Ar^F^), 1.37 (s, 18H, (C*H*_3_)_3_C), 1.06 (s, 18H, (C*H*_3_)_3_C), 0.12 (s, 6H, (C*H*_3_)_2_Si). ^13^C{^1^H} NMR (75 MHz, C_6_D_6_): δ 158.78 (3-pz*C*), 154.14 (5-pz*C*), 144.03 (*ipso*-Ar^F^*C*), 131.70 (q, *J*_CF_ = 33 Hz, *m*-Ar^F^*C*), 128.80 (m, *o*-Ar^F^*C*), 124.25 (q, *J*_CF_ = 270 Hz, *C*F_3_), 118.30 (sept, *J*_CF_ = 4 Hz, *p*-Ar^F^*C*), 102.95 (4-pz*C*), 74.08 (N_pz_–*C*H*)*, 32.35 ((CH_3_)_3_*C*), 32.21 ((CH_3_)_3_*C*), 30.70 (*C*H_3_)_3_C), 30.38 (*C*H_3_)_3_C), 25.67 (*C*H_2_–Ar^F^), −1.51 ((*C*H_3_)_2_Si). ^19^F NMR (282 MHz, C_6_D_6_): δ −63.32 (s, C*F*_3_). FTIR: cm^–1^ 2955 m, 2902w, 2869w, 1617w, 1536w, 1461w, 1367 m, 1340w, 1313w, 1274s, 1248w, 1235w, 1210w, 1168s, 1131s, 1066w, 1021w, 1000w, 919w, 904w, 891w, 845w, 832w, 814 m, 804 m, 730w, 707w, 681w, 646w, 615w, 501w. ESI-MS (+): *m*/*z* 657.376; calc. for [**1**H-H]^+^: *m/z* 657.378.

### Synthesis of **2**

The follow manipulations were performed in a glovebox. Proligand **1**H (2.645 g, 4.026 mmol) in THF (20 mL) was cooled to −78 °C in a glovebox cold well. A solution of *t-*BuLi in heptane (2.7 M, 1.65 mL, 4.46 mmol) added dropwise *via* syringe, resulting in an intensely yellow-brown solution. After stirring for 15 min at this temperature a suspension of FeCl_2_•*x*THF (prepared by stirring FeCl_2_ (766 mg, 6.04 mmol) in THF (5 mL) at room temperature (RT) overnight) was added *via* pipette. The reaction vessel was immediately removed from the cold well and allowed to come to RT with stirring. Stirring was continued for 2 h to afford a bright yellow solution with suspended black solids. Solvent was removed under reduced pressure, and the residue was extracted with C_6_H_6_ (40 mL) and filtered through a short pad of Celite (∼2 cm) in a 20 mL glass-fritted funnel. The Celite was washed with additional portions of C_6_H_6_ until the washings were colorless. The solvent was removed thoroughly under reduced pressure to leave a yellow-brown residue. *o*-Difluorobenzene (10 mL) was added to dissolve the bulk of the solids. The mixture was diluted with *n*-pentane (20 mL) and quickly filtered through a short pad of Celite (∼2 cm) in a 20 mL glass-fritted funnel. The Celite was washed with additional portions of *n*-pentane until the washings were colorless. The solvent was removed under reduced pressure to leave an oily yellow solid, which was triturated with *n*-pentane (20 mL) and collected by filtration. Washing with *n*-pentane (3 × 10 mL) gave **2** as a yellow, microcrystalline solid. Crystals suitable for XRD studies were obtained by slow evaporation of an Et_2_O solution at RT. Yield: 1.425 g (47%). RT magnetic moment (by Evans method in C_6_D_6_): 5.7 μ_B_. ^1^H NMR (300 MHz, C_6_D_6_): δ 110.46 (1H), 78.44 (1H), 66.53 (1H), 19.66 (9H, (C*H*_3_)_3_C), 13.91 (9H, (C*H*_3_)_3_C), 3.77 (3H (C*H*_3_)Si), 35.13 (12H, (C*H*_3_)Si + (C*H*_3_)_3_C), −40.95 (9H, (C*H*_3_)_3_C), −69.00 (1H), −93.23 (1H). ^19^F NMR (282 MHz, C_6_D_6_): δ −94.59 (s, C*F*_3_). NMR data was consistent with the expected *C*_1_ symmetry in solution. A presumably very broad resonance for the 2 × *o*-Ar^F^*H* was not observed. FTIR: cm^–1^ 2965 m, 2904w, 2867w, 1597w, 1535w, 1524w, 1460w, 1415w, 1364s, 1291w, 1271s, 1240m, 1206w, 1172m, 1160s, 1123s, 1096m, 1067m, 1054m, 1022w, 994w, 945m, 909w, 880w, 850m, 831m, 812m, 746m, 728w, 702m, 679m, 652w, 613w, 562w, 542w, 499w, 479w, 440w. UV–vis (THF): λ_max_ (nm) ε_max_ (M^–1^ cm^–1^) 324 (1.1 × 10^4^). Anal. calc. for C_34_H_49_ClF_6_FeNSi: C 54.66; H 6.61; N 7.50. Found: C 54.22; H 6.41; N 7.20.

### Synthesis of **3**

(C_5_Me_4_(SiMe_3_))_2_Ti (100 mg, 0.230 mmol) in C_6_H_6_ (2 mL) was added to solid **2** (100 mg, 0.134 mmol), and the suspension was gently agitated for several minutes to generate a homogeneous, intense orange-red solution. The mixture was filtered through a short pad of Celite (∼1 cm) in a glass pipette into a 1 dram vial with additional C_6_H_6_ (3 × 0.5 mL) used to assist the transfer. The solution was carefully concentrated to 0.5 mL (insufficient concentration results in markedly reduced yields), sealed, and left to stand at RT overnight. The supernatant was carefully removed from the resulting mass of black crystals, which were quickly washed with a single portion of (Me_3_Si)_2_O (1 mL). The wet crystals were suspended in *n*-pentane (2 mL) containing additional (C_5_Me_4_(SiMe_3_))_2_Ti (20 mg, 0.046 mmol) and transferred to a larger vial containing a stir bar. Further *n*-pentane (3 × 1 mL) was used to assist transfer of the remaining crystals. The red mixture was stirred for 1 h to afford a fine, crystalline suspension of the product, which was collected by filtration and washed with *n*-pentane (3 × 1 mL). Crystals of the centrosymmetric isomer suitable for XRD studies were obtained by concentrating the C_6_H_6_ solution described above to 1, rather than 0.5, mL and leaving the mixture for an additional 2 days. Crystals of the dissymmetric isomer were obtained by layering a concentrated THF solution of the complex with (Me_3_Si)_2_O at −30 °C for several days. Crystallization from THF-*n*-pentane also gave the dissymmetric isomer with crystals of poorer quality. NMR data obtained immediately upon redissolving these crystalline samples showed a slight excess of one or the other diastereomer, with re-equilibration to a ∼1:0.8 mixture after several hours (see the SI). Yield: 74–82 mg (76–84%). RT magnetic moment method (by Evans method in C_6_D_6_): 7.1 μ_B_. ^1^H NMR (300 MHz, C_6_D_6_): δ isomer *a* (dissym): 79.74 (1H), 59.03 (1H), 34.14 (3H, (C*H*_3_)Si), 26.96 (9H, (C*H*_3_)_3_C), 19.22 (9H, (C*H*_3_)_3_C), 5.01 (9H, (C*H*_3_)_3_C), −25.35 (1H), −26.95 (9H, (C*H*_3_)_3_C), −102.61 (1H); isomer *b* (sym.): 75.00 (1H), 64.57 (1H), 31.58 (3H, (C*H*_3_)Si), 25.90 ((C*H*_3_)_3_C), 22.70 (9H, (C*H*_3_)_3_C), −3.86 (9H, (C*H*_3_)_3_C), −13.34 (3H, (C*H*_3_)Si), −20.23 (9H, (C*H*_3_)_3_C), −67.06 (2H), −93.13 (1H). ^19^F NMR (282 MHz, C_6_D_6_): δ isomer *a* (dissym.): −98.32 (s, br, C*F*_3_); isomer *b* (sym.): −96.75 (s, C*F*_3_). FTIR: cm^–1^ 2956 m, 2907w, 2868w, 1592w, 1557w, 1526w, 1547w, 1457w, 1415w, 1395w, 1359s, 1288w, 1271s, 1241m, 1210w, 1157s, 1115s, 1093m, 1047w, 1019w, 992w, 943m, 878m, 85 m, 383m, 826m, 809m, 788w, 773w, 741w, 726w, 711w, 700m, 679m, 649w, 614w, 531s, 478m, 431m. The N≡N stretching mode for the dissymmetric isomer was too weak to be resolved. UV–vis (THF): λ_max_ (nm) ε_max_ (M^–1^ cm^–1^) 325 (2.2 × 10^4^), 450 (1.0 × 10^4^), 530 (6.2 × 10^3^), 671 (1.4 × 10^4^), 907 (4.5 × 10^3^). Comment on purity: perhaps owing to its extreme sensitivity to air and moisture, we have been unable to obtain an adequate elemental analysis for **3** despite several attempts. Solutions of **3** in C_6_H_6_ are homogenous, precluding the presence of inorganic salts.

### Synthesis of **4**

AdN_3_ (11.2 mg, 0.0632 mmol) in Et_2_O (1 mL) was added to a suspension of **3** (41.6 mg, 0.287 mmol) in Et_2_O (1 mL), resulting in immediate evolution of gas (N_2_). The mixture was gently agitated for several minutes to generate a homogeneous, orange-brown solution, which was filtered through a short pad of Celite (∼1 cm) in a glass pipette, and the Celite was washed with (Me_3_Si)_2_O (3 × 2 mL). The solution was concentrated under reduced pressure to ∼4 mL and stored at −30 °C for several hours. The resulting brown needles were collected by filtration and washed with (Me_3_Si)_2_O (3 × 1 mL). Crystals suitable for XRD studies were obtained by layering a concentrated THF solution of the complex with (Me_3_Si)_2_O at −30 °C for several days. The complex decayed slowly in solution at RT, and so, spectroscopic measurements were made as rapidly as possible. Yield: 42.0 mg (85%). RT magnetic moment method (by Evans method in C_6_D_6_): 4.2 μ_B_. ^1^H NMR (300 MHz, C_6_D_6_): δ 111.40 (1H), 49.26 (6H, Ad*H*), 40.02 (1H), 30.24 (1H), 27.65 (3H, Ad*H* or (C*H*_3_)Si), 21.83 (3H, Ad*H* or (C*H*_3_)Si), 21.39 (3H, Ad*H* or (C*H*_3_)Si), 18.74 (9H, (C*H*_3_)_3_C), 14.46 (3H, Ad*H* or (C*H*_3_)Si), 13.13 (9H, (C*H*_3_)_3_C), −13.58 (3H, Ad*H* or (C*H*_3_)Si), −19.83 (1H), −22.67 (9H, (C*H*_3_)_3_C), −40.53 (9H, (C*H*_3_)_3_C). ^19^F NMR (282 MHz, C_6_D_6_): δ −73.22 (s, C*F*_3_). NMR data was consistent with the expected *C*_1_ symmetry in solution. Presumably very broad resonances for C*H*–Fe and 2 × *o*-Ar^F^*H* were not observed. FTIR: cm^–1^ 2959m, 2897m, 2843m, 1594w, 1557w, 1459w, 1446w, 1415w, 1394w, 1360s, 1295w, 1272s, 1243m, 1219w, 1209w, 1162s, 1123s, 1096w, 1068m, 1025w, 995w, 941 m, 908w, 888w, 849w, 834w, 809w, 760w, 749w, 727w, 705w, 681w, 652w, 490w, 440w. UV–vis (THF): λ_max_ (nm) ε_max_ (M^–1^ cm^–1^) 313 (1.7 × 10^4^), 499 (2.3 × 10^3^), 672 (1.3 × 10^3^). Anal. calc. for C_44_H_64_F_6_FeN_5_Si•0.3((CH_3_)_3_Si)_2_O: C 60.47; H 7.69; N 7.70. Found: C 60.20; H 7.39; N 7.39.

### Computational Details

All calculations were carried out using revision 5.0.2 of the ORCA suite of programs.^38^ DFT calculations made use of the “TIGHTSCF” convergence criteria; unless stated otherwise, default settings were used for all other methods.

Models of **2**, **3**, and **4** (hereafter **2′**, **3′**, and **4′**) were constructed from the crystallographically determined coordinates, truncating the bulky 3,5-di-*t*-butylpyrazolyl substituents to 3,5-dimethylpyrazolyl, which we anticipated would accurately model the electronics of the true substituents,^39^ while reducing computational cost. For **4′**, we additionally truncated the *N*-adamantyl substituent to *N*-methyl. While the heavy atoms of each model were thus fixed, the positions of the H-atoms were allowed to relax along the ground-state potential energy surface for **2′** (*M*_S_ = 2), **3′** (*M*_S_ = 3), and **4′** (*M*_S_ = 3/2) using the BP86 exchange-correlation functional along with the def2-TZVP basis set.^40−42^

Using these models, single-point calculations were carried out on **2′**, **3′**, and **4′** using the meta-GGA exchange-correlation functional TPSS including 0% (TPSS), 10% (TPSSh), and 25% (TPSS0) exact Hartree–Fock (HF) exchange.^43−45^ Scalar relativistic effects were included using the zeroth order regular approximation (ZORA).^46^ The recontracted ZORA-def2-TZVPP basis was employed for all atoms heavier than C as well as the C(H) moiety directly bound to the Fe center, whereas the smaller ZORA-def2-TZVP(−f) basis was employed for all other C and H atoms.^47^ For all atoms, the general-purpose segmented all-electron relativistically contracted auxiliary Coulomb fitting basis (SARC/J) was employed, which is a decontraction of the def2/J basis developed by Weigend.^48^ Calculations including HF exchange were accelerated through the RIJCOSX approximation.^49^

For single-point calculations of **3′** and **4′**, we generated low-spin, broken-symmetry (BS) determinants by first converging high-spin determinants (for **3′**, *M_S_* = 4; for **4′**, *M_S_* = 5/2), and subsequently exchanging the α and β spin density matrix elements on the nitrogenous ligands via the FlipSpin feature of ORCA, and reconverging a low-spin determinant (for **3′**, *M_S_* = 3; for **4’***M_S_* = 3/2). For the analysis of DFT wavefunctions in terms of localized molecular orbitals, we employed the intrinsic bond orbital (IBO) method developed by Knizia and implemented in ORCA.^50^ Alternatively, BS determinants were analyzed in terms of the corresponding orbital transformation.^51^

In addition to DFT studies, we investigated the magnetic properties of **4′** using a multireference *ansatz* (CASSCF). Multireference calculations employed the recontracted versions of Dunning’s correlation-consistent basis sets tailored for use with the second-order Douglas–Kroll–Hess (DKH2) Hamiltonian, which was used to account for scalar relativistic effects.^52^ The basis sets were of double-ζ quality (cc-pVDZ-DK) on the C and H atoms, and triple-ζ quality (cc-pVTZ-DK) on the heavier atoms as well as the C(H) moiety directly bound to the Fe center.^53,54^ To accelerate these calculations, the RIJK approximation was used on combination with the cc-pVTZ/JK auxiliary Coulomb/exchange fitting basis for all atoms,^55^ excluding the Fe atom, for which an auxiliary basis was constructed on the fly using the AutoAux feature of ORCA;^56^ all auxiliary bases were fully decontracted. CASSCF wavefunctions were converged to a tolerance of 1 × 10^–7^ E_*h*_.

Following convergence of the CASSCF reference, a second-order *N*-electron valence perturbation theory (NEVPT2) calculation was performed to better account for the effects of dynamic correlation on the energies of the computed states;^57^ NEVPT2 calculations used the strongly contracted variant parameterized in ORCA.^58^ The magnetic properties (*i.e.*, *D*- and *g*-tensors) of the *S* = 3/2 ground state of **4′**, including the effects of both spin-orbit and spin–spin coupling, were computed *via* the effective Hamiltonian approach using the NEVPT2-corrected state energies.^59^

## Results and Characterization

From surveying the array of reported “heteroscorpionates”,^60^ the ligand precursor **1**H, which contains two pyrazoles and a relatively acidic benzylic group (Scheme 1), was designed and prepared according to established synthetic procedures (see the Experimental Section). Deprotonation of **1**H using *t*-BuLi followed by *in situ* metalation using FeCl_2_ gave the corresponding high-spin Fe(II) complex (**1**)FeCl (**2**, Scheme 1) as a bright yellow, crystalline solid in 40–50% yield.

**Figure 2:**
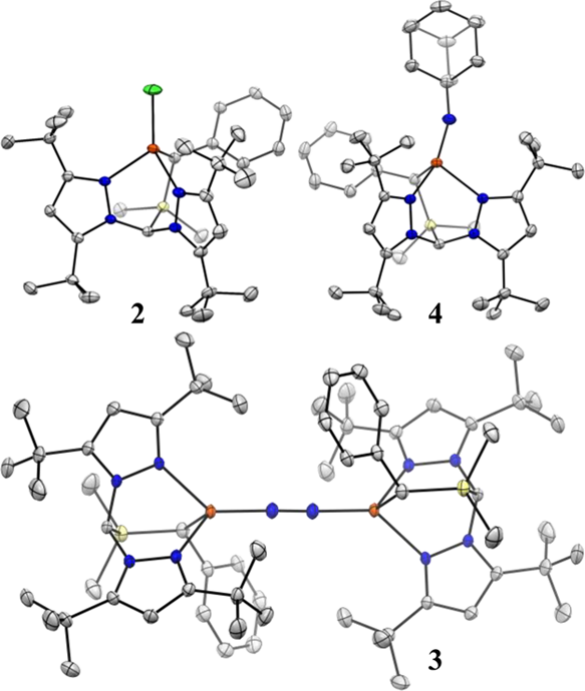
Thermal ellipsoid plots (50%) of **2**, **3**, and **4**. Orange, blue, yellow, and gray ellipsoids represent Fe, N, Si, and C, respectively. Hydrogen atoms, solvent molecules, and CF_3_ groups are omitted for clarity. All molecules crystallized in the *P*-*1* space group

Attempts to reduce **2** using conditions typical for generating N_2_ complexes, such as alkali metals or derivatives thereof, invariably gave intractable mixtures. Fortuitously, reduction using the bulky titanocene (Cp^±^)_2_Ti^II^ (Cp^±^ = C_5_Me_4_(SiMe_3_)) gave the dark-red, formally Fe(I), bridging N_2_ complex [(**1**)FeN]_2_ in ∼80% yield (**3**, Scheme 1). Given the stability of **3**, it is not clear why reduction of **2** using alkali metals proved unproductive. One possibility is that the reduction of **3** outcompetes reduction of **2**, leading to over-reduction and decomposition. We are unable to completely rule this out, although careful monitoring of the reaction mixtures revealed complex mixtures formed immediately upon addition of reductant; indeed, only metallic Li in Et_2_O generated detectable amounts of **3***in situ*. We speculate that the initial product of one-electron reduction, *i.e.*, [(**1**)FeCl][M] (M = Li, Na, K), decomposes *via* a low barrier pathway before N_2_ is able to coordinate. Attempts to reduce **3** using alkali metals gives complex mixtures from which we have been unable to isolate any one component. The chiral C-donor results in two diastereomers for **3**; these interconvert quickly in solution but could both be crystallized using different solvent combinations and showed near-identical metrics and spectroscopic properties (see Experimental Section and the SI). This interconversion could be a result of C-decoordination and epimerization and/or monomerization to afford the presumably *S* = 3/2 terminal N_2_ adduct [(**1**)Fe(N_2_)]; at this stage, we are unable to completely rule out either possibility. Spectroscopically, **3** is typical of low-coordinate, N_2_-bridged Fe complexes,^61−65^*e.g.*, the solution state magnetic moment of 7.1 μ_B_ is consistent with the usual well-isolated *S* = 3 ground state. Likewise, the solid state structure of **3** (Figure 2) is reminiscent of other Fe(I)_2_(μ-η^1^:η^1^-N_2_) dimers,^62,64,66,67^*i.e.*, roughly tetrahedral Fe sites with a near linear M–N_2_–M subunit. The Fe–N and N–N distances of 1.804(1) and 1.185(2) Å, respectively, suggest some Fe–N multiple bond character and substantial N–N bond weakening.^68^

With the isolation of **3**, we were curious to ascertain if ligand **1** could support higher-valent, Fe–N multiply bonded species akin to those implicated as N_2_ reduction intermediates.^4^ Thus, 1-adamantyl azide was added to **3**, resulting in rapid gas evolution and formation of the orange-brown, terminal Fe(III)-imido complex (**1**)Fe(NAd) (**4**, Scheme 1). In the absence of air and moisture, **4** is quite robust, with <10% decomposition to a mixture of species upon standing in C_6_D_6_ solution at RT for 24 h. This is remarkable, given that addition of strong L-type donors to 3-coordinate Fe(III) imidos typically results in rapid H-atom abstraction reactivity at N_imido_.^69,70^

Unlike **2** and **3**, the Fe center for **4** deviates appreciably from tetrahedral geometry. The imido ligand for **4** tilts away from the Fe–C_basal_ vector, inducing partial planarization of the C-N_pz1_-N_imido_-Fe moiety; the angle between the Fe–N_imido_ bond vector and the C-N_pz1_-Fe plane is 24° (*c.f.* 55° for strictly tetrahedral). While the pair of Fe–N_pz_ distances differ by less than 0.02 Å in both **2** (*d*_Fe–Npz1_ = 2.109(2) and *d*_Fe–Npz2_ = 2.116(2) Å) and **3** (*d*_Fe–Npz1_ = 2.153(1) and *d*_Fe–Npz2_ = 2.173(1) Å), in **4**, a dramatic desymmetrization occurs, with one Fe–N_pz_ distance nearly 0.15 Å longer than the other: *d*_Fe–Npz1_ = 2.084(1) *c.f. d*_Fe–Npz2_ = 2.231(1) Å. Indeed, the latter is the longest reported Fe–N_pz_ interaction for a ≤4-coordinate Fe center.^71^ This is all the more conspicuous considering the contraction of the other Fe–N_pz_ distance, which follows a nearly linear trend across the series **3** → **2** → **4,** as expected with the increasing formal oxidation state of the Fe center. The extent to which the long Fe–N_pz_ interaction in **4** can be considered a “bond”, then, is ambiguous. We opt, therefore, to describe the weakly bound pyrazole as effectively hemilabile. Notably, the free energy of binding 4-*t*-butylpyridine to the 3-coordinate, trigonal planar imido complex (NacNac)Fe(NAd) (NacNac = 1,3-diketiminate) is very low—*ca.* 1 kcal mol^–1^ at RT—and a DFT-calculated structure of this four-coordinate species is reminiscent of the solid-state structure of **4**, including an exceptionally long Fe–N_pyridine_ distance.^72^ The Fe–N_imido_ distance for **4** of 1.717(1) Å is much shorter, however, than that calculated for (NacNac)Fe(NAd)(pyridine) (1.76 Å) and only slightly longer than reported for (NacNac)Fe(NAd).^69,70^ The extent to which this relates to the relative thermal stability of **4** is unclear; (NacNac)Fe(NAd)(pyridine) has proven to be too reactive to isolate.

**Figure 3:**
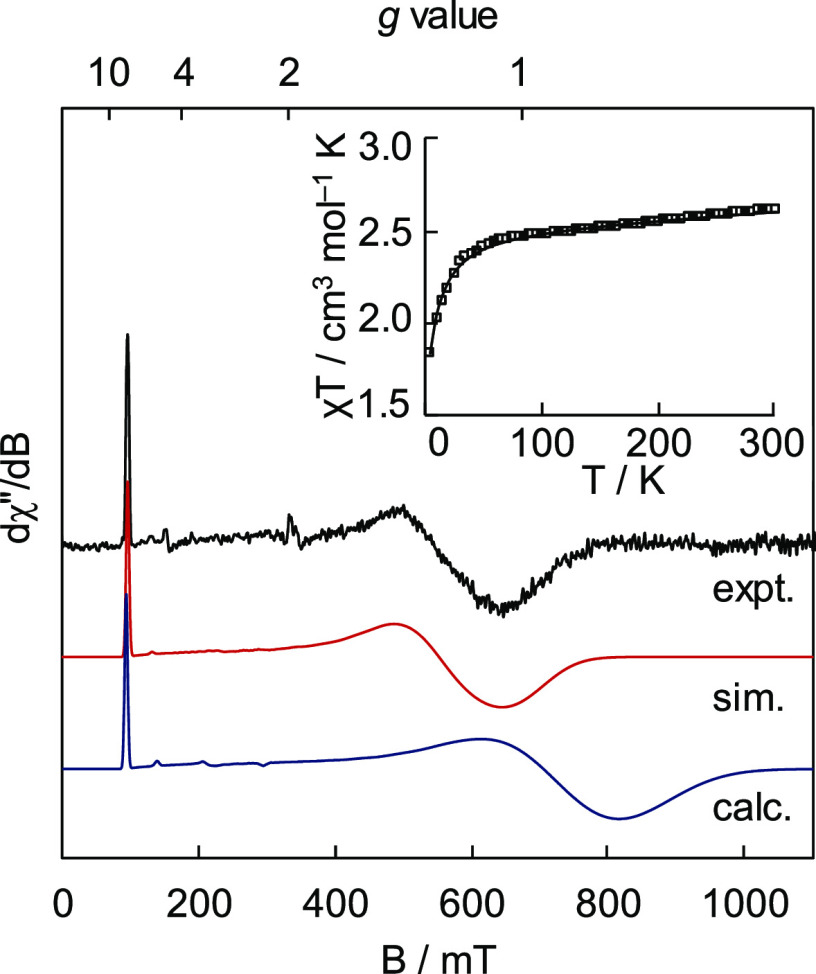
CW EPR spectrum of **4**, its simulation (red), and CASSCF-calculated spectrum (blue). The experimental spectrum was recorded at 9.37 GHz and 0.25 mW power in a toluene glass at 15 K. A least-squares smoothing function has been applied to reduce noise at high field. The background signal due to the spectrometer cavity has been subtracted. Simulation parameters: *D* = −30 cm^–1^, *E/D* = 0.19; *g* = 2.39, 2.03, 2.03 (*g*_iso_ = 2.15); *D*-strain (cm^–1^) = 0, 1.19, 0.228; linewidth (mT) = 5. Details for the calculated spectrum are given in the main text and the SI. Inset: SQUID magnetometry data (squares) and simulation (line) recorded on a solid sample of **4** at 10,000 Oe applied field. The simulation parameters are identical to those for the EPR data but with added first-order spin-orbit coupling of 3.1 cm^–1^.

In solution, the magnetic moment of 4.2 μ_B_ is consistent with **4** adopting an intermediate (*S* = 3/2) spin ground state; SQUID magnetometry confirms this assignment with no evidence for spin-crossover behavior or appreciable thermal population of excited states (Figure 3, inset). The SQUID data could be well-simulated using an *S* = 3/2 spin Hamiltonian using parameters identical to those used to simulate EPR data obtained for **4** (*vide infra*). The EPR spectrum of **4** (Figure 3) recorded in frozen glass at 15 K reveals an approximately axial signal with *g*_obs_ ≈ 6.9, 1.2, reminiscent of those reported for trigonal planar, intermediate-spin (NacNac)Fe(III) imido complexes.^69,70^ For **4**, this spectrum does not readily conform to an *S* = 3/2 rhombogram assuming *g_x_* = *g_y_* = *g_z_* = *g_e_*, implying substantial spin-orbit coupling as a result of low-lying excited states. This is supported by CASSCF calculations (*vide infra*), with the EPR spectrum for **4** well reproduced in silico (Figure 3). High-field/variable temperature EPR experiments are required to provide precise zero-field splitting (ZFS) parameters for **4**; however, reasonable estimates have been obtained from simulation of the presented experimental data. The calculated axial ZFS parameter *D* of −30 cm^–1^ was used directly, and no reasonable simulations could be obtained using positive *D*. At this stage, we are unable to fully delineate contributions to the effective *g*-tensor due to rhombicity in the ZFS (*E/D*) and *g*-anisotropy arising from spin-orbit coupling. Consequently, *g*_iso_ cannot presently be determined with accuracy, although all reasonable simulations give *g*_iso_ > 2. That said, *g*_1_ (2.39; calcd. 2.42) is invariant to rhombicity and can be stated with confidence. Ultimately, *E*/*D* was simulated as 0.19, slightly higher than the calculated value of 0.15, as this provided *g* values of the roughly axial symmetry predicted *in silico*. Pronounced broadening in the *g*_2_ and *g*_3_ features can be attributed to a small distribution in rhombicity (“*D*-strain”) and is similarly featured in the calculated spectrum.

Overall, then, structural and EPR data supports a description of **4** as only nominally four-coordinate and best described as three-coordinate with an additional weak Fe–N_pz_ interaction. The hemilabile pyrazole donor thus allows Fe to readily adopt the (ostensibly) low-coordination numbers preferred as Fe–N covalency increases at the unique site. The absence of complete dissociation of one pyrazole, as might be expected if **1** contained only one such donor, also prevents Fe from adopting a *bona fide* trigonal planar geometry and thus modulates the large structural distortion this would entail.

## Calculations

### Complex **3**

A range of arguments have been presented in the literature to explain the mechanism of N_2_ activation in Fe(I)_2_(μ-η^1^:η^1^-N_2_) complexes, from covalent π-backbonding^61^ to complete Fe-to-N_2_ electron transfer, *i.e.*, two high spin Fe(II) centers (*S* = 2) antiferromagnetically coupled to a bridging triplet N_2_^2–^ (*S* = 1), giving rise to the observed septet spin state (*S* = 2 + 2 – 1 = 3).^73^ The collective orbital picture for the primary Fe–N π-interactions as provided by broken-symmetry (BS) DFT calculations (Figure S17) suggest that the Fe(II)–(N_2_^2–^)–Fe(II) resonance structure does contribute to the ground-state description of **3′**. However, the relative weights of Fe(I)–(N_2_^0^)–Fe(I) and Fe(II)–(N_2_^2–^)–Fe(II) contributors (and, indeed, Fe(I)–(N_2_^•–^)–Fe(II) ↔ Fe(II)–(N_2_^•–^)–Fe(I)) remain uncertain.

### Complex **4**

**Figure 4:**
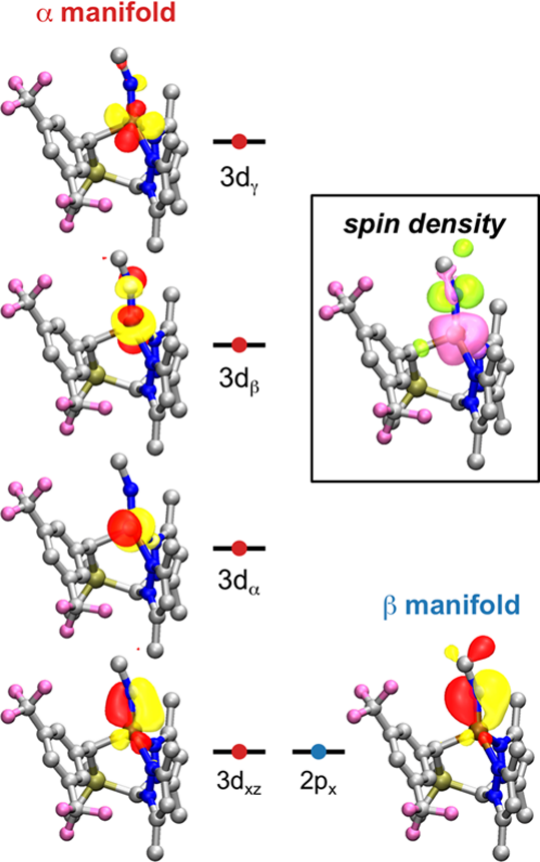
Frontier magnetic orbitals from a BS DFT calculation of **4′** using 25% HF (TPSS0; isovalue = 0.05 a.u.). Inset: spin densities from the same calculation. Pink surfaces show α density, while green surfaces show β density (isovalue = 0.005 a.u.).

Based on spectroscopic and computational evidence, four-coordinate Fe imidos in weak ligand fields have been characterized as high-spin metal centers antiferromagnetically coupled to one-electron oxidized imido radical anions.^72,74^ To investigate whether a similar description is appropriate for **4**, we first performed a series of BS DFT calculations. The magnetic orbitals obtained from a BS DFT calculation of the *S* = 3/2 state of **4′** using 25% HF exchange and the corresponding spin density plot are presented in Figure 4. In addition to the three expected open shells—which are predominantly admixtures of the 3d_*x*^2^__–*y*^2^_, 3d_*z*^2^_, and 3d_*yz*_ orbitals—a pair of magnetic orbitals is found with overlap significantly smaller than unity. These orbitals can be identified as belonging to the “in-plane” Fe–N_imido_ π-interaction (where “in-plane” here refers to the *xz* plane defined by Fe, N_imido_, and C_alkyl_). The DFT calculations thus suggest a description of **4′** as a high-spin Fe(II) center (*S* = 2) antiferromagnetically coupled to an open-shell nitrene radical anion (NR^•−^, *S* = 1/2), giving rise to the observed quartet spin state (*S* = 2 –  = ). This spin structure can be gleaned from the DFT-calculated spin density, where the nominal imido ligand possesses significant, anisotropic, negative spin density, originating from the singly occupied 2p_*x*_ orbital (Figure 4, inset). This is quantitatively dependent on the degree of HF exchange employed (see the SI for discussion).

To obtain a more precise ground-state electronic structure description of **4′**, we performed a state-specific CASSCF calculation of the *S* = 3/2 ground spin state. For this purpose, we restricted ourselves to a CAS(7,6) space including (i) the three primarily 3d-based SOMOs, (ii) the bonding/anti-bonding pair constituting the in-plane Fe–N_imido_ π interaction, and (iii) the orthogonal N_imido_ π lone pair (see the SI for rationale and calculated orbitals). The DFT calculations presented above suggested a description of **4′** as a high-spin Fe(II) center (*S* = 2) antiferromagnetically coupled to a NR^•–^ (*S* = 1/2) ligand. In the language of configuration–interaction, this would correspond to significant diradical character in the “in-plane” Fe–N_imido_ π-bond. Such character can be quantified *via* the diradical index^75^
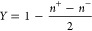
where *n^±^* is the natural orbital occupation number (NOON) for the bonding (antibonding) interaction. Thus, *Y* = 0 corresponds to a normal covalent bond, whereas *Y* = 1 corresponds to a pure singlet diradical interaction (*i.e.*, the two involved electrons are spatially separated and antiferromagnetically coupled to give a singlet). On the basis of the NOONs for the 3d_*xz*_ ± 2p_*x*_ orbitals (1.57 and 0.44) obtained from the state-specific CAS(7,6) calculation of the *S* = 3/2 ground state, the diradical index of the “in-plane” Fe–N_imido_ π interaction is *Y* = 0.44. In a simplified representation, we can use this analysis to ascribe to the *S* = 3/2 ground state a valence-bond like resonance between a “standard” covalent description, [Fe(III)(NR^2–^)] (56%), and an antiferromagnetically coupled pair [Fe(II)(NR^•–^)] (44%). This depiction is analogous to that proposed for (NacNac)Fe(NAd)(pyridine)^72^ as well as one-electron oxidized congeners of **4′**,^74^ although **4′** displays substantially weaker coupling between Fe and N_imido_ compared to the latter. We can use the sextet–quartet splitting (2674 cm^–1^) computed with an enlarged CAS(9,7) active space to extract the HDVV exchange-coupling constant, *J* = −2674/5 = −535 cm^–1^ (see the SI for details). This is in good agreement with the DFT calculation using 25% HF (−541 cm^–1^). Moreover, a Löwdin spin population analysis suggests that the spin density calculated at the 25% HF level is in best agreement with the state-specific CAS(7,6) results (see Table S4). Unlike for **3**, we can assess the quality of our multireference calculations *via* comparison with the experimental EPR data (Figure 3; see the SI for details).

## Discussion

**Figure 5:**
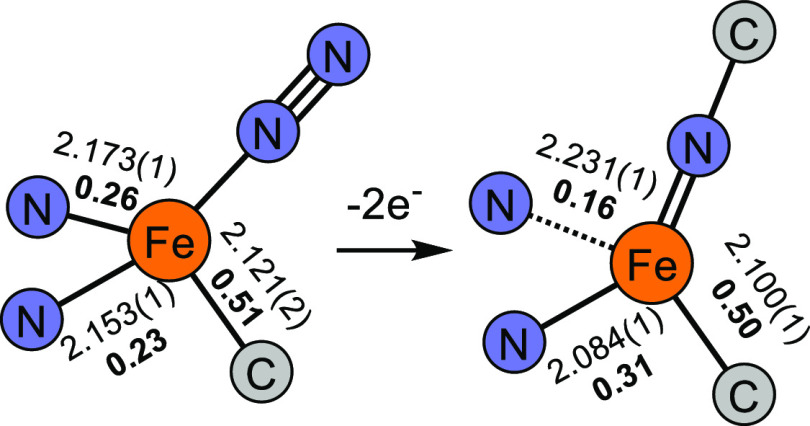
Structural changes accompanying the conversion of **3** → **4**. The numbers alongside bonds are distances in Å; those in bold are MBOs.

It is instructive to scrutinize the structural distortions that accompany the transformation from **3**—featuring relatively reduced Fe centers and a π-acidic terminal ligand—to **4**—featuring a relatively oxidized Fe center and a π-basic terminal ligand (Figure 5). Our prior work has demonstrated that low-valent Fe centers in weak, *C*_3_-symmetric ligand fields are well-suited to the binding and activation of π-acids such as N_2_.^64,76^ Accordingly, the two Fe–N_pz_ interactions for each Fe center in **3** are effectively equivalent (Δ*d*_Fe–Npz_ = 0.02 Å), and are calculated to have very similar low Mayer bond orders (MBOs) of 0.23 and 0.26, reflecting largely ionic bonding. In contrast, reported intermediate-spin Fe(III) imidos exhibit small to no thermodynamic preference for binding a fourth ligand (*vide supra*). This manifests as partial decoordination of one pyrazole donor in **4**; indeed, the calculated Mayer bond order (MBO) for the lengthened Fe–N_pz_ (0.16) interaction is halved in comparison with the other (0.31). Throughout **2**–**4**, the Fe–C_alkyl_ bond is largely unperturbed (Δ*d*_Fe–C_ = 0.02 Å). We posit that this observation can be attributed to the enhanced covalency of the Fe–C_alkyl_ interaction relative to the Fe–N_pz_ interactions. Indeed, while the MBOs calculated for the Fe–C_alkyl_ interaction of **3** (0.51) and **4** (0.50) remain small in absolute terms, they are substantially larger than those for the Fe–N_pz_ interactions. Thus, the transformation of **3** → **4** illustrates how a combination of static and fluxional interactions between a metal and a supporting ligand can accommodate changes in π-bonding at the unique ligand site.

## Conclusions

This work demonstrates that a strongly σ-donating NNC heteroscorpionate ligand is able to support Fe bound by both π-acidic N_2_ and π-basic imido ligands. The different geometries at Fe induced by switching between N_2_ and RN^2–^ groups is modulated by a static, relatively covalent Fe–C_alkyl_ interaction and a hemilabile pyrazole donor. This suggests possible applications of our ligand in a variety of metal-mediated, multi-electron processes. Fe–S distances in Fe_4_S_4_ clusters are known to undergo substantial deformation upon redox events.^77^ This and our findings reported herein suggest the possibility that Fe−μ_3_S, rather than Fe–C, lability may facilitate substrate binding and reduction at the active Fe site(s) of FeMoco.

## Supporting Information Available

Spectroscopic data and additional computational details (PDF)
