# Supplementary material for: Four-Coordinate Fe N2 and Imido Complexes Supported by a Hemilabile NNC Heteroscorpionate Ligand
Source: Inorg Chem. 2022 Jul 27;61(31):12318–26. doi: 10.1021/acs.inorgchem.2c01656 (PMC9367695; doi:10.1021/acs.inorgchem.2c01656)
Supplement: Supplementary file 1 — ic2c01656_si_001.pdf [file ic2c01656_si_001.pdf]

Supporting Information for:

Four-coordinate Fe N_2 and Imido Complexes Supported by a Hemilabile NNC Heteroscorpionate Ligand

**Alex McSkimming[1] and Niklas B. Thompson[2]*

[1]Department of Chemistry, Tulane University, New Orleans, LA 70118; [2] Argonne National Laboratory, Lemont, IL 60439.

*amcskimming@tulane.edu

Contents

Spectroscopic Data	S3
Structure of $\mathbf{3}_{\mathbf{dissym}}$	S11
Additional Computational Details	S12
Additional Details for **3'**	S12
Additional Details for **4'**	S15
Multireference Description of **4'**	S17
Excited State Calculations of **4'**	S21
Evaluating Metal-Ligand Covalency for **2'**, **3'**, and **4'**	S24
References	S26
Calculated Coordinates	S27

Spectroscopic Data

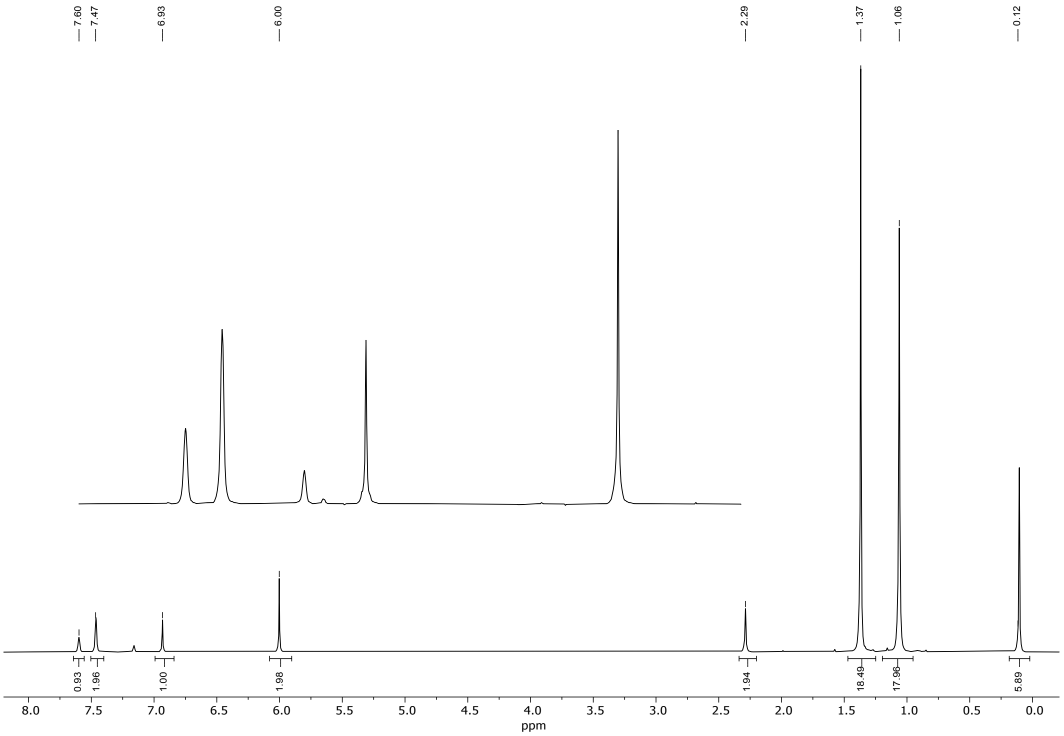

Figure S1. ^{1}H NMR spectrum of **1**H recorded in C_6D_6 at 300 MHz. Aromatic region, inset.

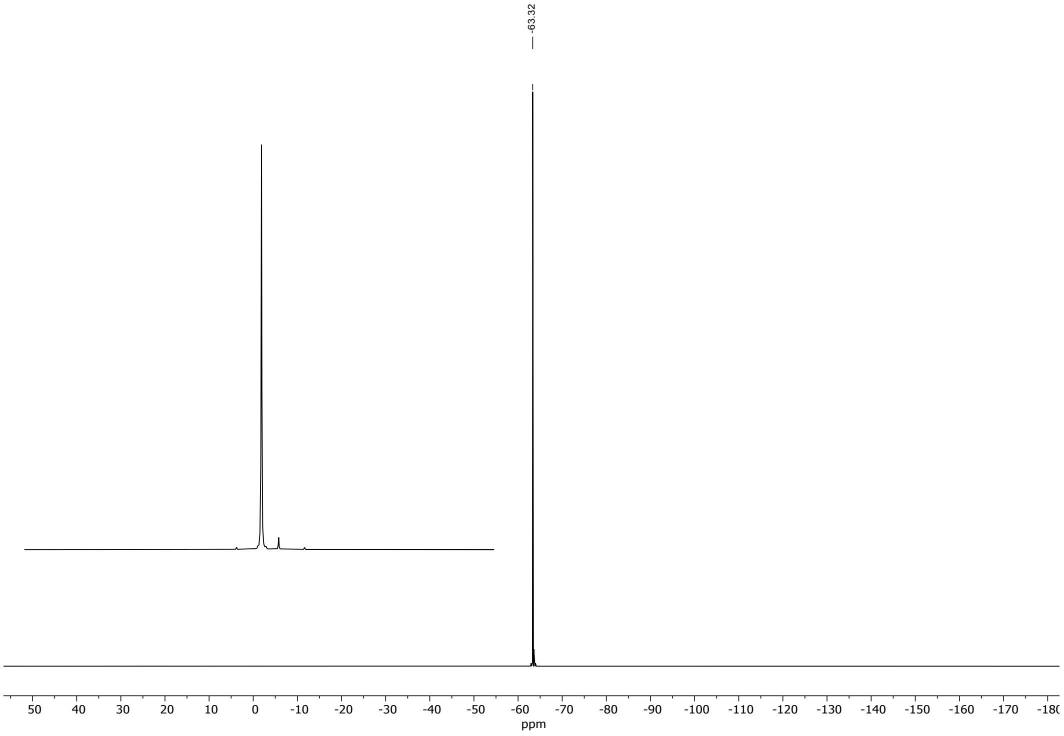

Figure S2. ^{19}F NMR spectrum of **1**H recorded in C_6D_6 at 282 MHz. Close-up, inset.

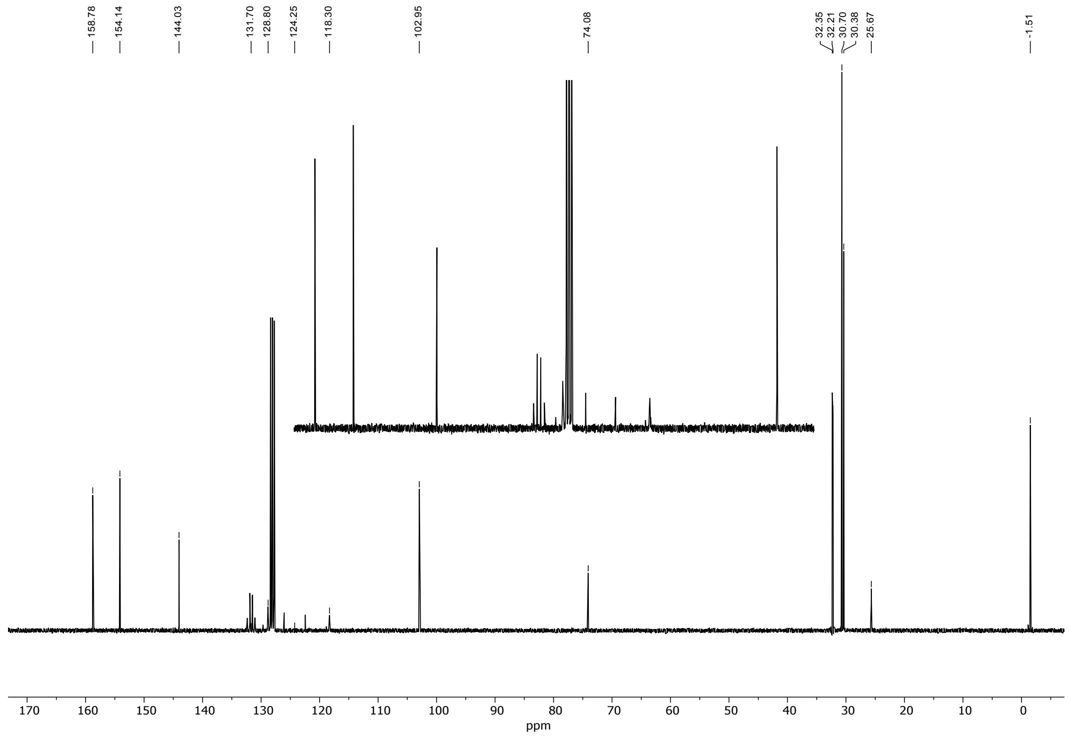

Figure S3. $^{13}C\{^{1}H\}$ NMR spectrum of **1**H recorded in C_6D_6 at 75 MHz. Aromatic region, inset.

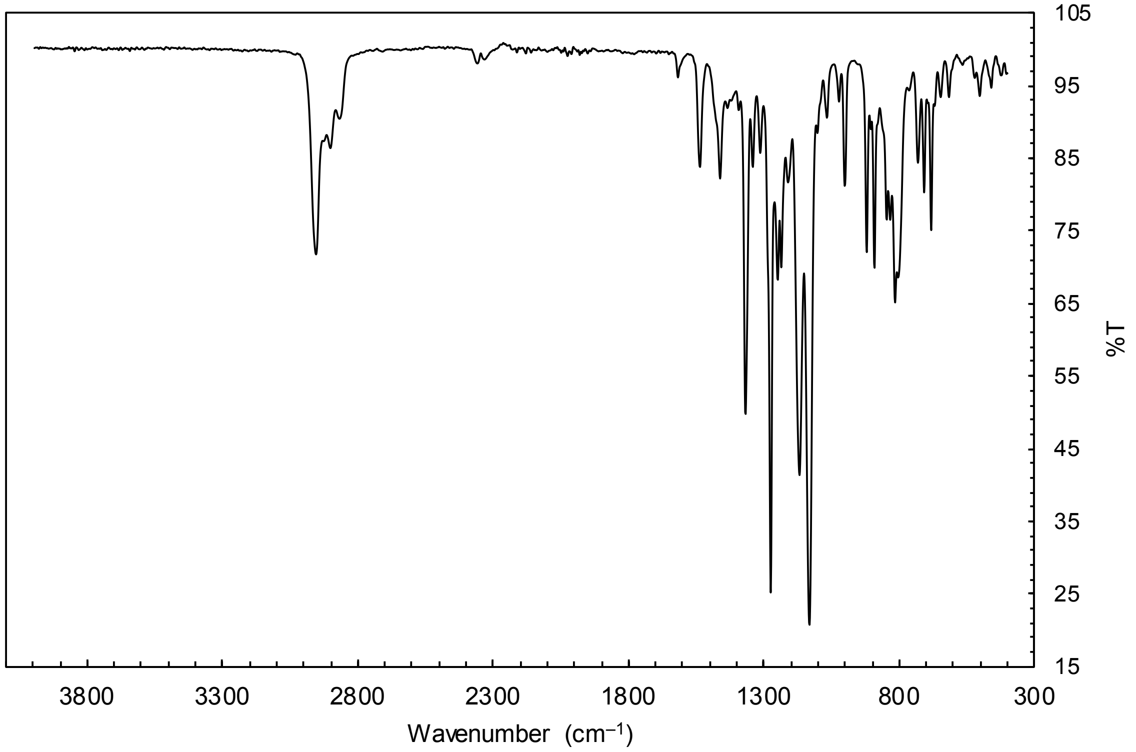

Figure S4. FTIR spectrum of **1**H.

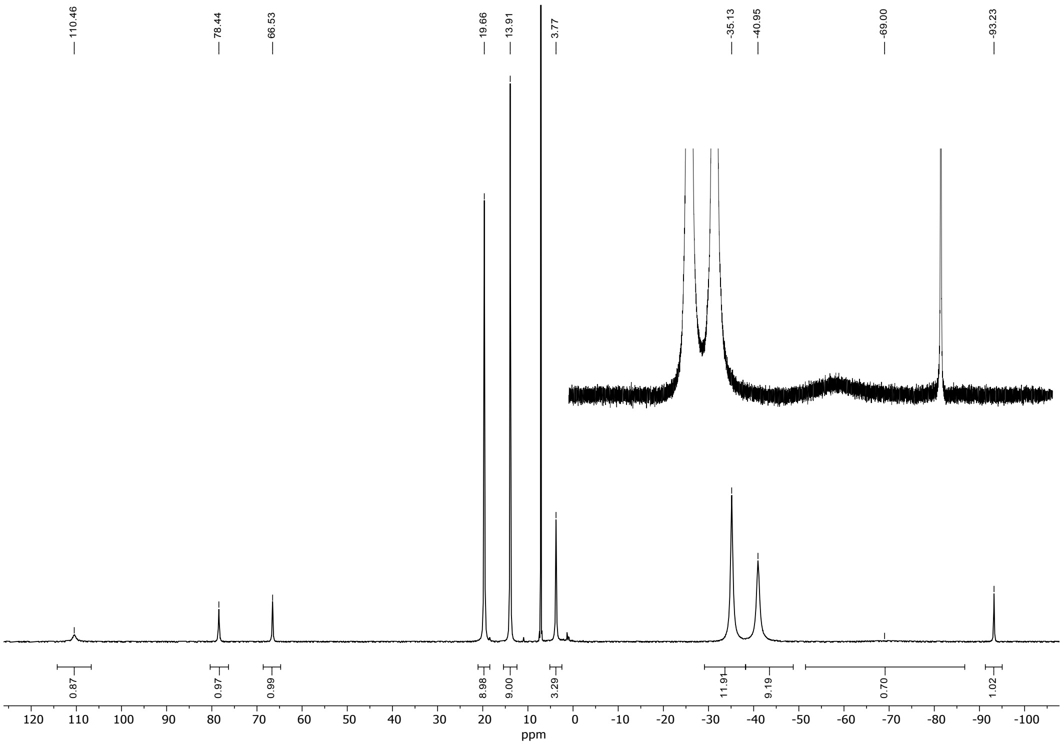

Figure S5. ^{1}H NMR spectrum of **2** recorded in C_6D_6 at 300 MHz. δ –10 to –120 region, inset.

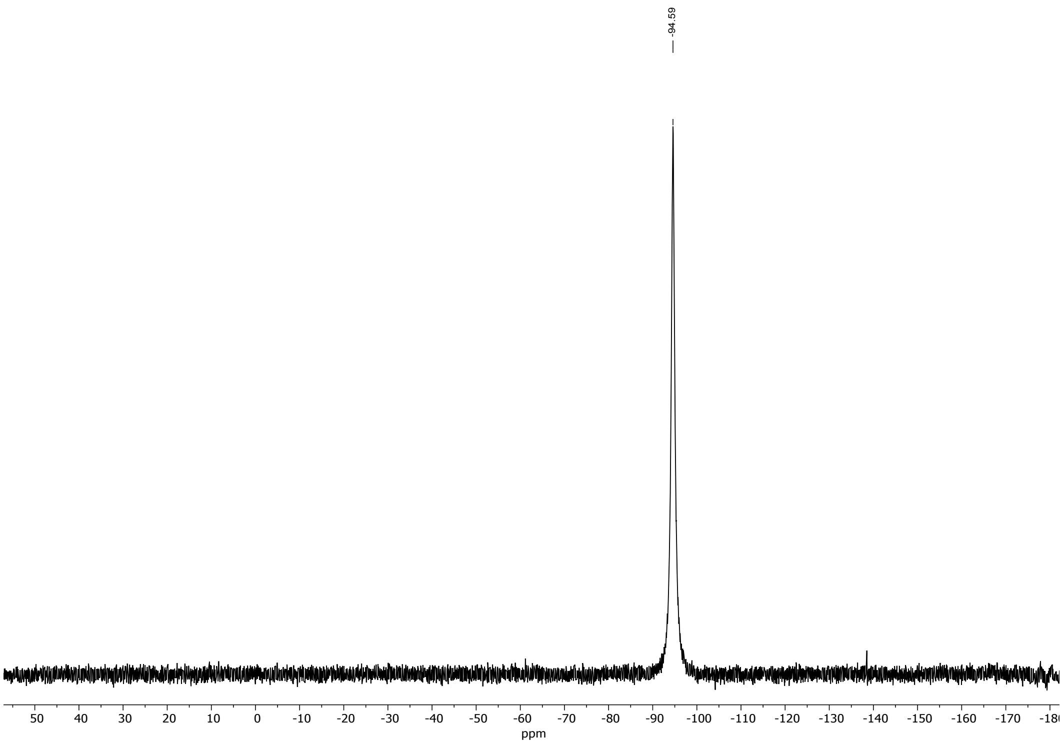

Figure S6. ^{19}F NMR spectrum of **2** recorded in C_6D_6 at 282 MHz.

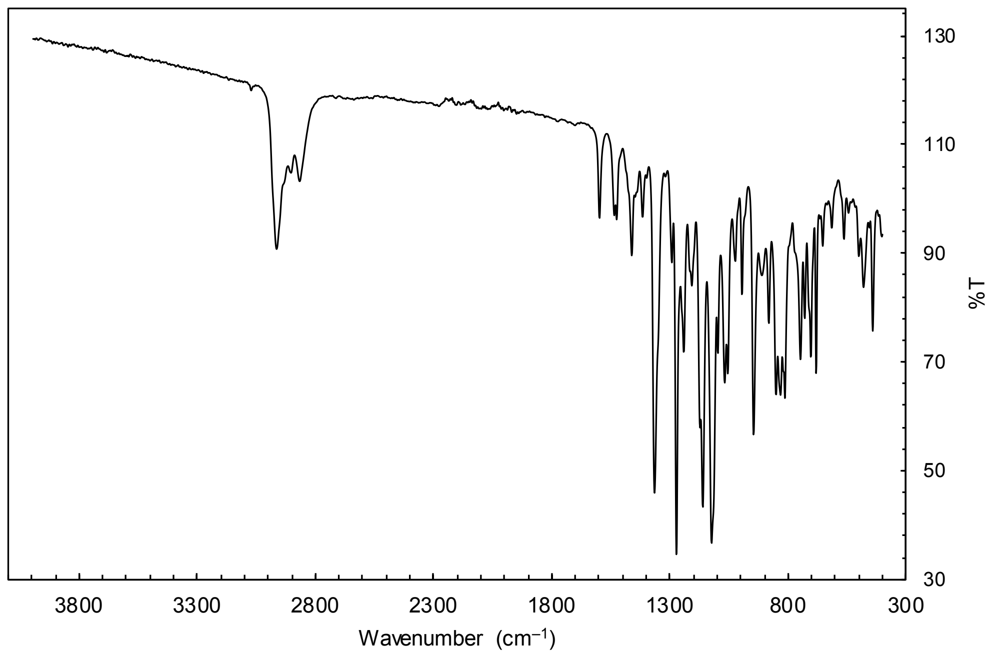

Figure S7. FTIR spectrum of **2**.

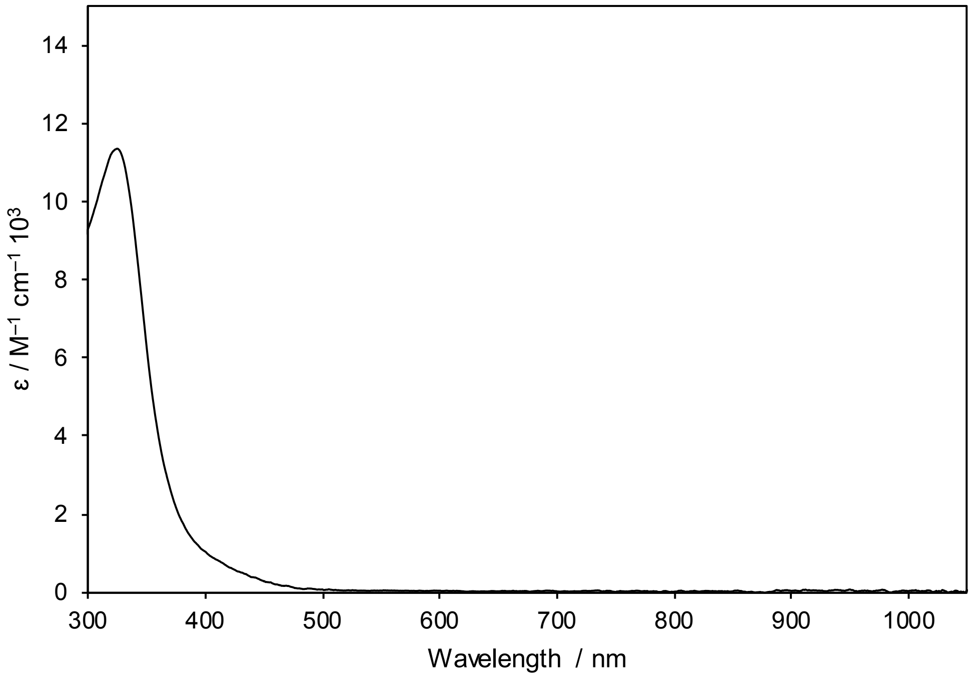

Figure S8. UV-Vis spectrum of **2** in THF.

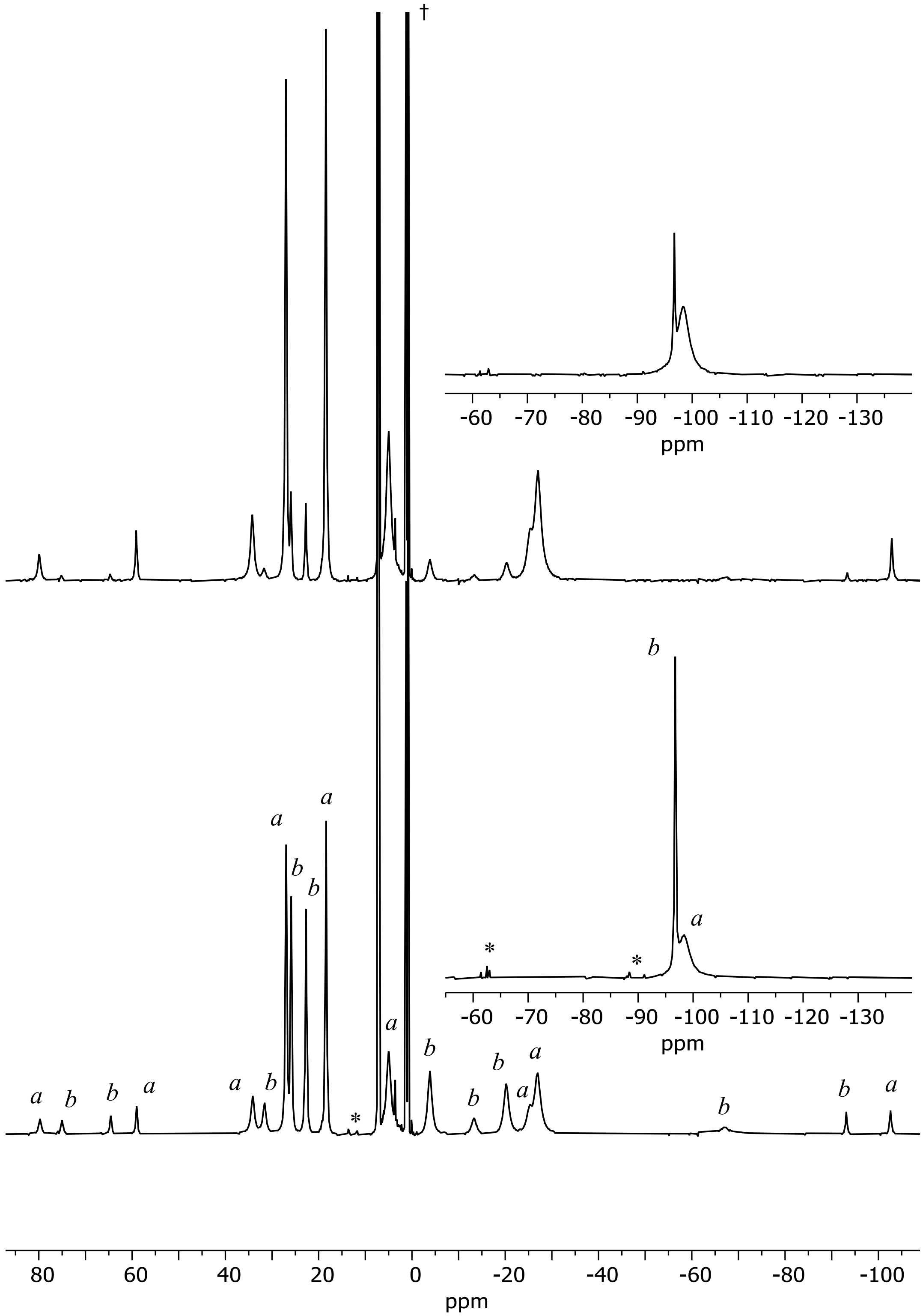

Figure S9. ^{1}H and ^{19}F NMR (inset) spectra of **3** recorded in C_6D_6 at 300 MHz recorded for a crystalline sample of the dissymmetric isomer immediately (top) and after 4h (bottom). Peaks assigned to the different diastereomers are labelled '*a*' (dissym.) or '*b*' (sym.). * indicates impurities due to thermal degradation, † n-pentane of crystallization.

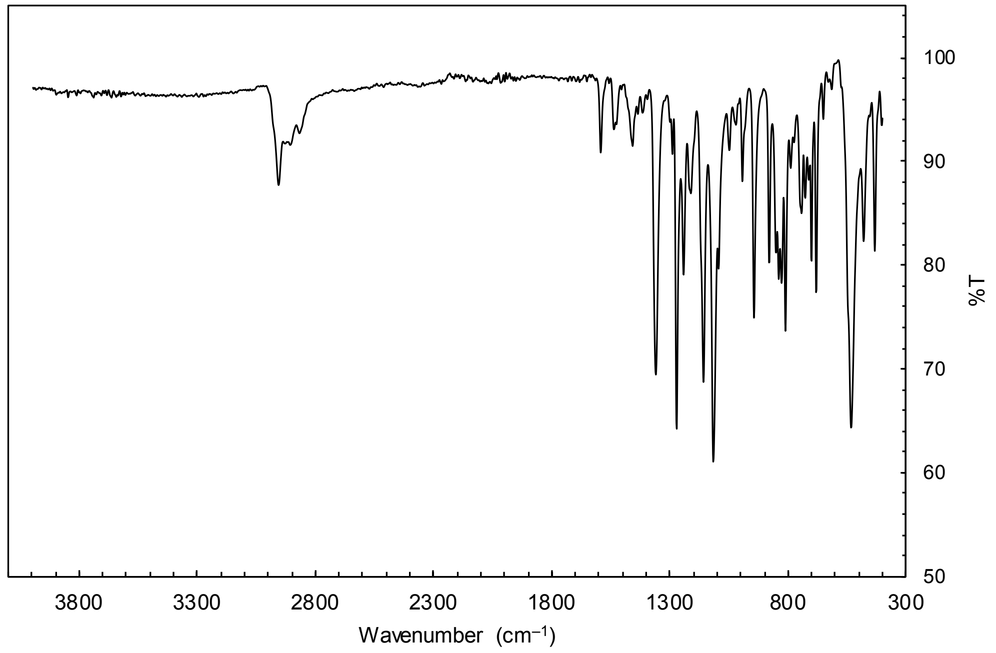

Figure S10. FTIR spectrum of **3**.

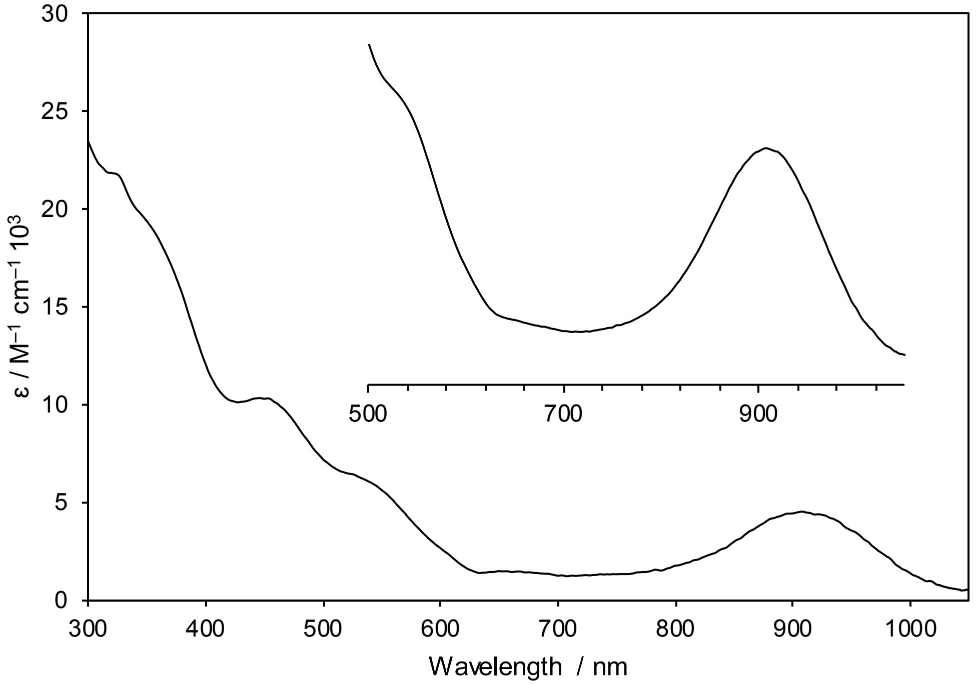

Figure S11. UV-Vis spectrum of **3** in THF (at 3-fold concentration, inset).

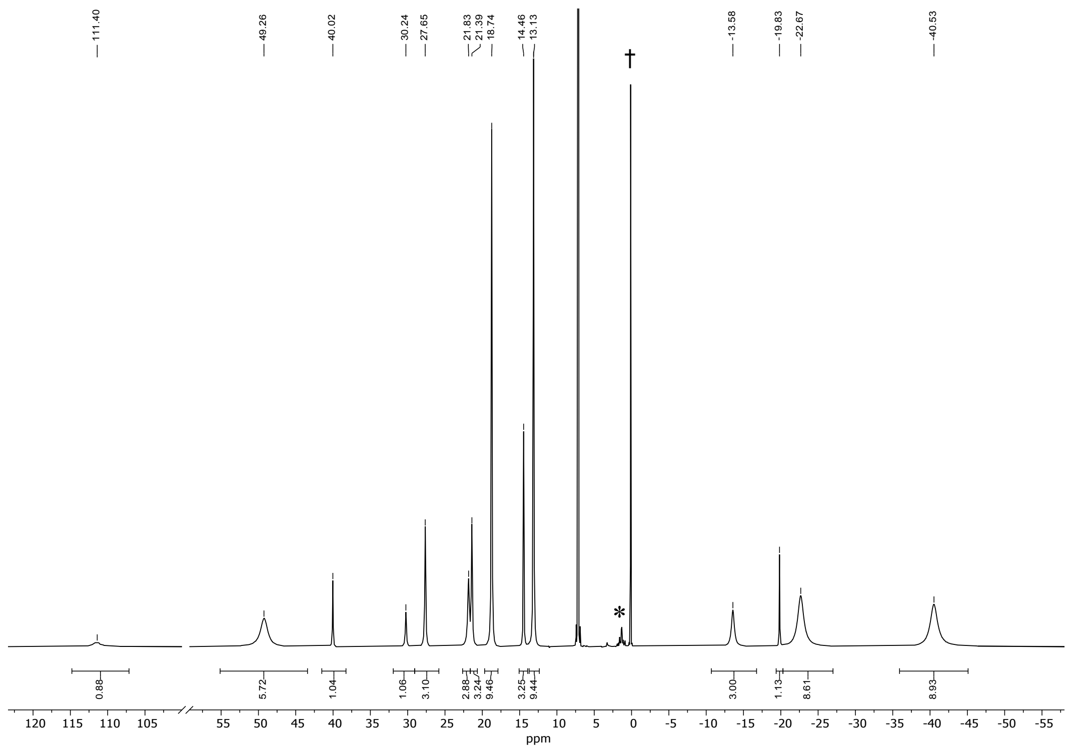

Figure S12. ^{1}H NMR spectrum of **4** recorded in C_6D_6 at 300 MHz. * indicates diamagnetic impurities due to thermal degradation, accounting for ~1% of the mixture by integration. † $(Me_3Si)_2O$.

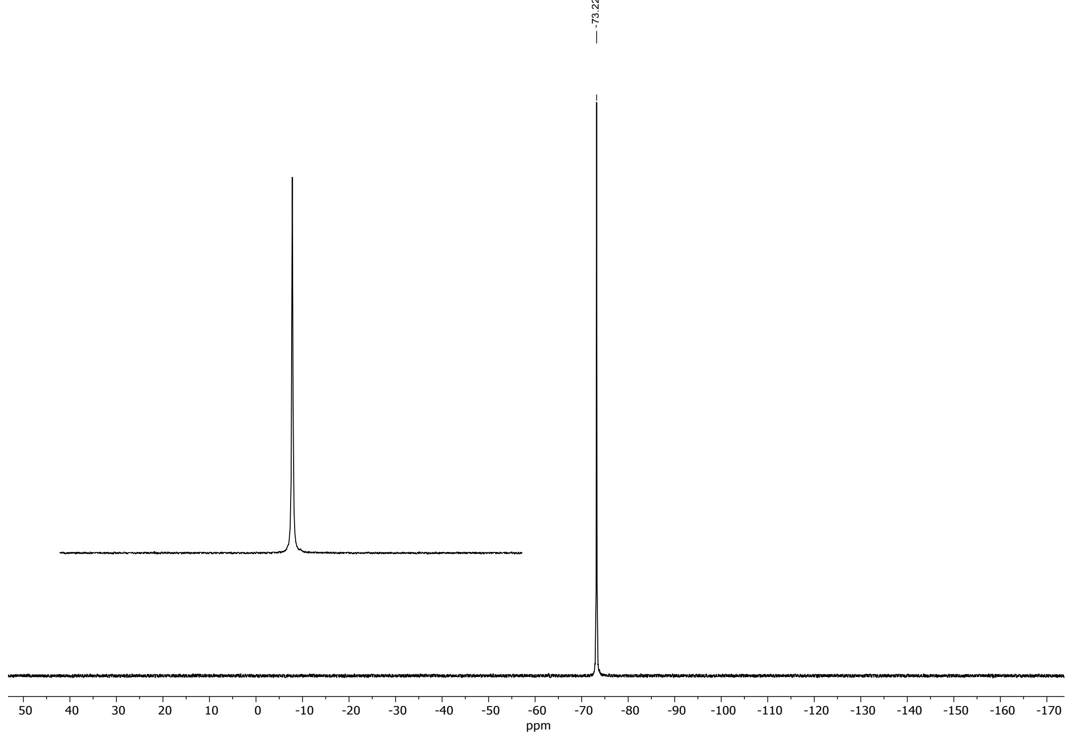

Figure S13. ^{19}F NMR spectrum of **4** recorded in C_6D_6 at 282 MHz. Close-up, inset.

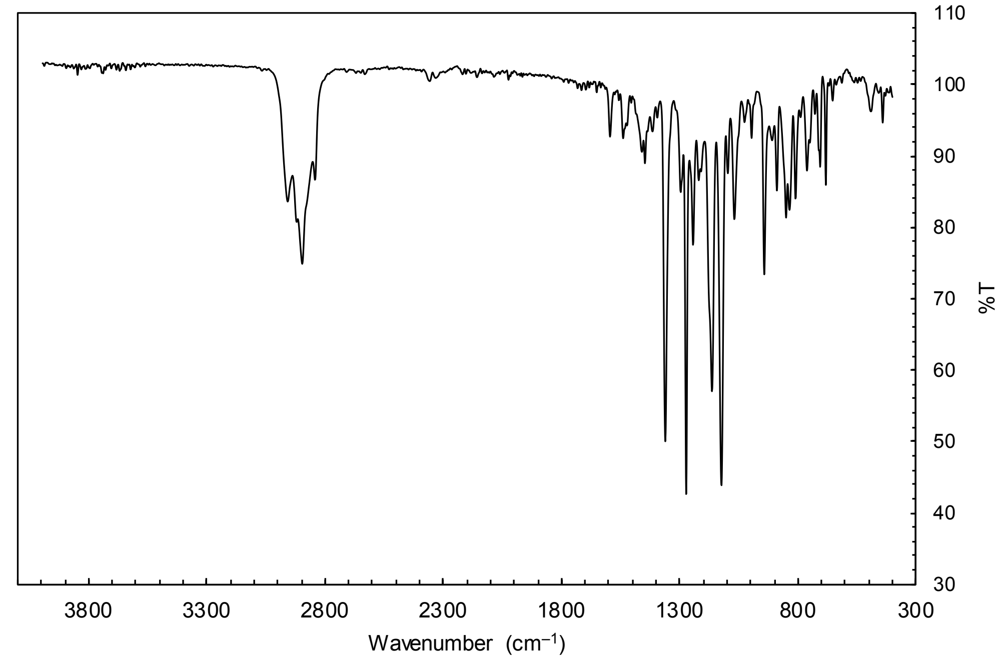

Figure S14. FTIR spectrum of **4**.

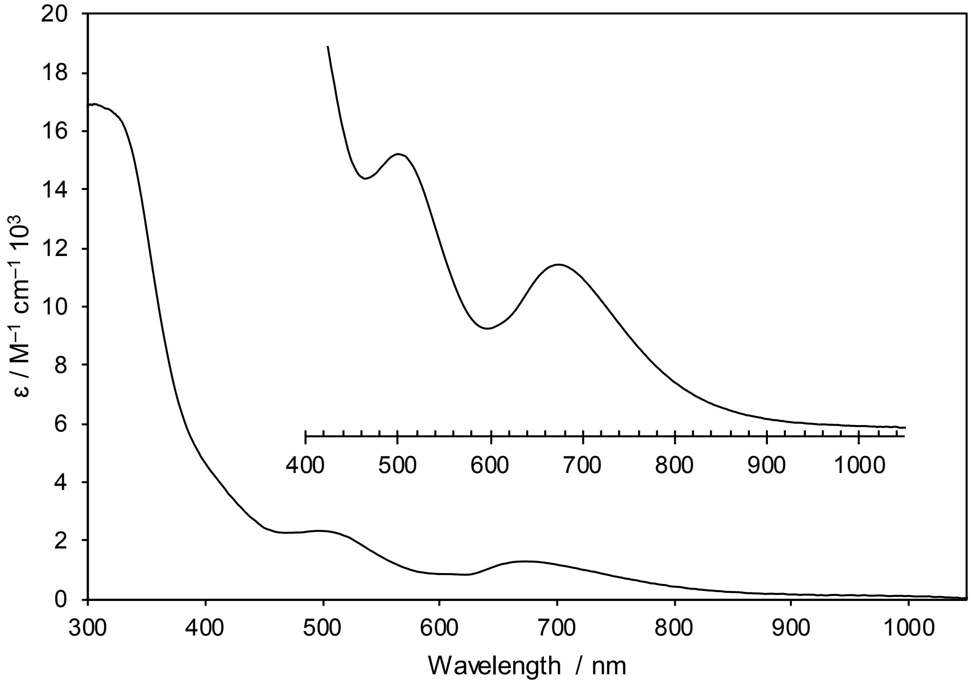

Figure S15. UV-Vis spectrum of **4** in THF (at 3-fold concentration, inset).

Structure of 3_{dissym}

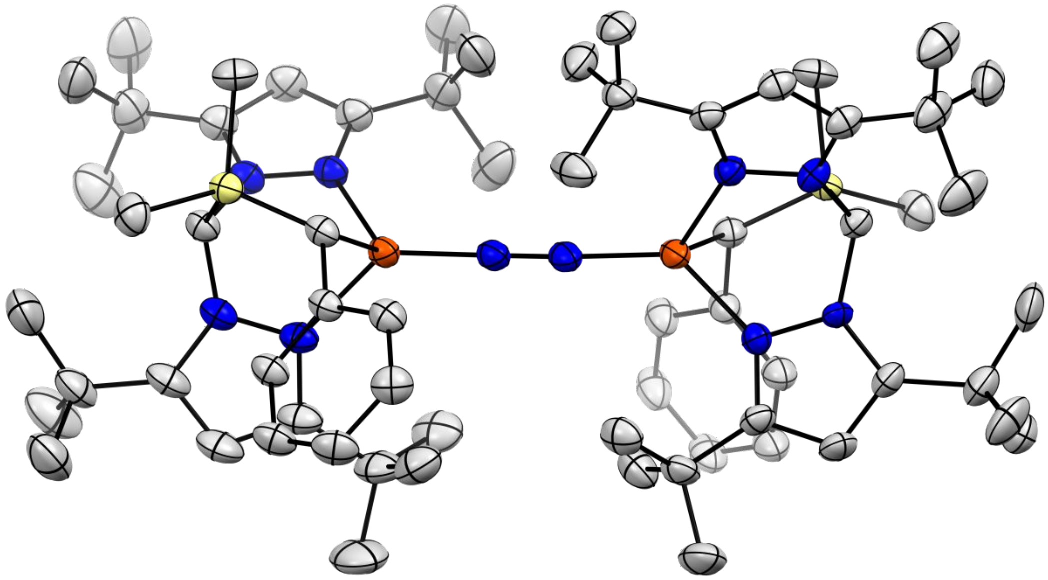

Figure S16. Thermal ellipsoid plot (50%) of the dissymmetric isomer of **3**. Orange, blue, yellow, and gray ellipsoids represent Fe, N, Si, and C, respectively. Hydrogen atoms, solvent molecules and CF_3 groups are omitted for clarity.

Additional Computational Details

Additional Details for 3'

As computational support for the various descriptions of bonding in $Fe(I)_2(m\text{-}h^1\text{:}h^1\text{-}N_2)$ complexes has largely been provided by DFT, the discussion is complicated by the well-documented dependence of metal-ligand covalency on the degree of HF exchange used in the functional.[1] Consequently, without careful calibration of the DFT functional, or the use of projection techniques, the spin density on the N_2 fragment is *not* a quantitative proxy for the extent to which it has been reduced: pure functionals (0% HF) tend to overestimate metal-ligand covalency (overly delocalized), while the HF description tends to underestimate the metal-ligand covalency (overly localized).

To illustrate this point, we present the results of three calculations of the $M_S = 3$ state of **3'** with the same exchange-correlation functional (TPSS), but with increasing amounts of HF exchange (0, 10, and 25%). The magnetic orbitals obtained from a calculation using 25% HF are shown in Figure S17A. In addition to the six open shells expected from two ferromagnetically-aligned Fe(I) centers, there are two pairs of magnetic orbitals with overlap significantly less than one. These can be identified as the two orthogonal Fe–(N_2)–Fe π-interactions, involving the two π* orbitals of the N_2 fragment and their symmetry-adapted Fe_2 d_π group orbitals. The character of this BS determinant thus suggests the spin-coupled picture described above, i.e., two high spin Fe(II) centers ($S = 2$) antiferromagnetically coupled to a central triplet N_2^{2-} ($S = 1$), giving rise to the observed septet spin ($S = 2 + 2 - 1 = 3$). As expected, the overlap between the N_2^{2-} π* orbitals and the metal d_π orbitals—and, hence, the description of the bonding situation along the continuum from a normal, covalent π-backbond to a diradical, exchanged-coupled interaction— is a function of the % HF included in the calculation (Table S01).

We can analyze the BS solutions in terms of a previously discussed three-spin Heisenberg-Dirac-Van Vleck (HDVV) Hamiltonian,[2] in which we couple the Fe spins (S_a and S_b) into an intermediate spin quantum number S_{ab}, which we couple with the N_2-derived spin (S_c) to yield the total spin,

$$\hat{S}_a + \hat{S}_b = \hat{S}_{ab}$$
$$\hat{S}_{ab} + \hat{S}_c = \hat{S}$$

Assuming S_{ab} to be a good quantum number, this reduces to the standard two-spin coupling HDVV Hamiltonian,

$$\hat{H} = -2J\hat{S}_{ab} \cdot \hat{S}_c$$

We can use Yamaguchi's formula to estimate the exchange-coupling constant from the DFT-calculated high-spin (M_S = 4) and BS states (M_S = 3),[3]

$$J = \frac{-(E_{\mathrm{HS}} - E_{\mathrm{BS}})}{\langle \hat{S}^2 \rangle_{\mathrm{HS}} - \langle \hat{S}^2 \rangle_{\mathrm{BS}}}$$

The values of J as a function of % HF are collected in Table S01. We also report the predicted splitting between the S = 3 and S = 4 states (−8J). The results are quite similar to those obtained for the related β-diketiminate-ligated system, where the authors predict the first excited spin state to occur at *ca.* 14,000 cm^{-1}.[2]

Table S01: Spin expectation values, corresponding orbital overlaps, and exchange-coupling results for **3**'

% HF	$\langle \hat{S}^2 \rangle_{\mathrm{BS}}$	$\langle 3\mathrm{d}_{\pi,\mathrm{xz}} \vert \pi^*_{\mathrm{xz}} \rangle$	$\langle 3\mathrm{d}_{\pi,\mathrm{yz}} \vert \pi^*_{\mathrm{yz}} \rangle$	***J* (cm^{-1})**	**−8*J* (cm^{-1})**
0	12.1343	0.98	0.98	−1647.49	13,179.12
10	12.1583	0.97	0.97	−1398.56	11,188.48
25	12.9168	0.79	0.70	−1496.12	11,968.96

Importantly, we note that the ambiguities in the DFT calculations are essentially quantitative. For example, the spin densities computed as a function of % HF are qualitatively similar: negative toroidal spin density on each N atom of the N_2 unit, arising from occupation of the two orthogonal π* orbitals (Figure S17B).

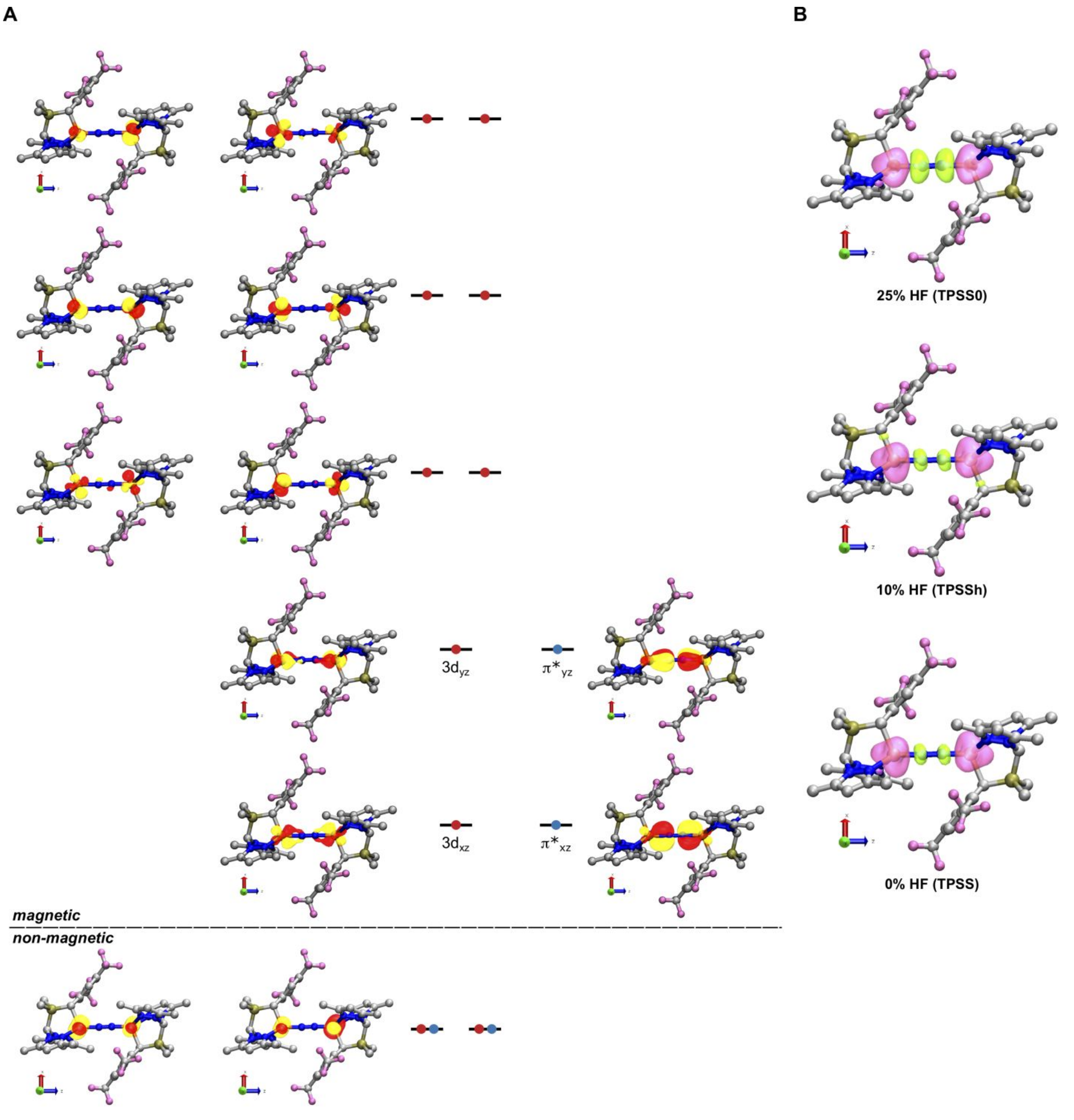

Figure S17. (A) Magnetic orbitals from a BS DFT calculation of **3'** using 25% HF (TPSS0; isovalue = 0.05 a.u.). (B) Spin densities obtained from BS DFT calculations of **3'**. Pink surfaces show α density while green surfaces show β density (isovalue = 0.005 a.u.).

Additional Details for 4'

The Fe–N spin-coupling interaction can be quantified via the standard Heisenberg–Dirac–Van Vleck Hamiltonian (HDVV),

$$\hat{H} = -2J\hat{S}_{\mathrm{Fe}} \cdot \hat{S}_{\mathrm{NR}}$$

again using Yamaguchi's formula to estimate the exchange-coupling constant from the DFT-calculated high-spin ($M_S = 5$) and BS states ($M_S = 3$).[3] As expected (*vide supra*), the strength of the antiferromagnetic coupling is directly related to the overlap of the non-trivial magnetic orbitals, which, in turn, is inversely proportional to the % HF used in the calculation (Table S02). This can also be visualized in the evolution of the DFT-calculated spin-densities as one transitions from 25 to 0% HF (Figure S18). For concreteness, we also tabulate the sextet–quartet energy gap ($-5J$) predicted by the phenomenological HDVV Hamiltonian, which is useful for comparison with the multireference calculations herein and in the main text.

Table S02: Spin expectation values, corresponding orbital overlaps, and exchange-coupling results for **4'**

**% HF**	$\langle \hat{S}^2 \rangle_{\mathrm{BS}}$	$\langle 3\mathrm{d}_{\mathrm{xz}} \vert 2\mathrm{p}_{\mathrm{x}} \rangle$	**J (cm^{-1})**	**−5J (cm^{-1})**
0	3.8584	0.95	−971.59	4,857.95
10	4.0068	0.87	−707.77	3,538.85
25	4.2840	0.70	−540.89	2,704.45

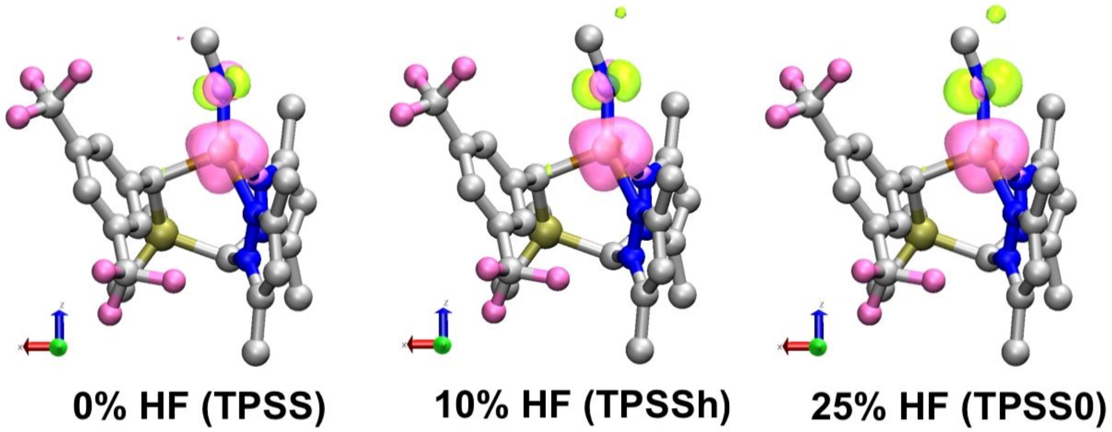

Figure S18. Spin densities obtained from BS DFT calculations of **4'**. Pink surfaces show α density while green surfaces show β density (isovalue = 0.01 a.u.).

Multireference Description of 4'

Active Space Selection

We tested several active space compositions for **4'**. On the basis of the single-reference results obtained from DFT calculations, the smallest space considered was CAS(5,5) (*i.e.*, 5 electrons in 5 orbitals), consisting of the three primarily 3d-based SOMOs of **4'** in addition to the bonding/anti-bonding orbitals corresponding to the "in-plane" Fe-N_{imido} π-interaction ($3d_{xz} \pm 2p_x$). Next, we expanded to a CAS(7,6) space, including the orthogonal pair of imido π-symmetry electrons ($2p_y$). Finally, to properly account for excited sextet states, we included the final Fe 3d orbital, for a CAS(9,7) active space. Figure S19 shows the relative energies of the ground doublet, quartet, and sextet states of **4'** from a 3-root, state-averaged CASSCF (SA-CASSCF) calculation as a function of the active space composition, demonstrating the importance of static correlation effects in the "out-of-plane" Fe-N_{imido} π-interaction for a proper description of the doublet excited state, and inclusion of all five 3d orbitals for a proper description of the sextet excited state. We note that the ground spin state is predicted to be S = 3/2 for all active space compositions. Although the CAS(9,7) space includes a nominally doubly-occupied $3d_{xy}$ orbital, a second-shell $3d'_{xy}$ orbital in an expanded CAS(9,8) active space was essentially unoccupied (NOON of 0.008) after optimization in a test SA-CASSCF calculation averaging over the first five doublet, quartet, and sextet states.[4]

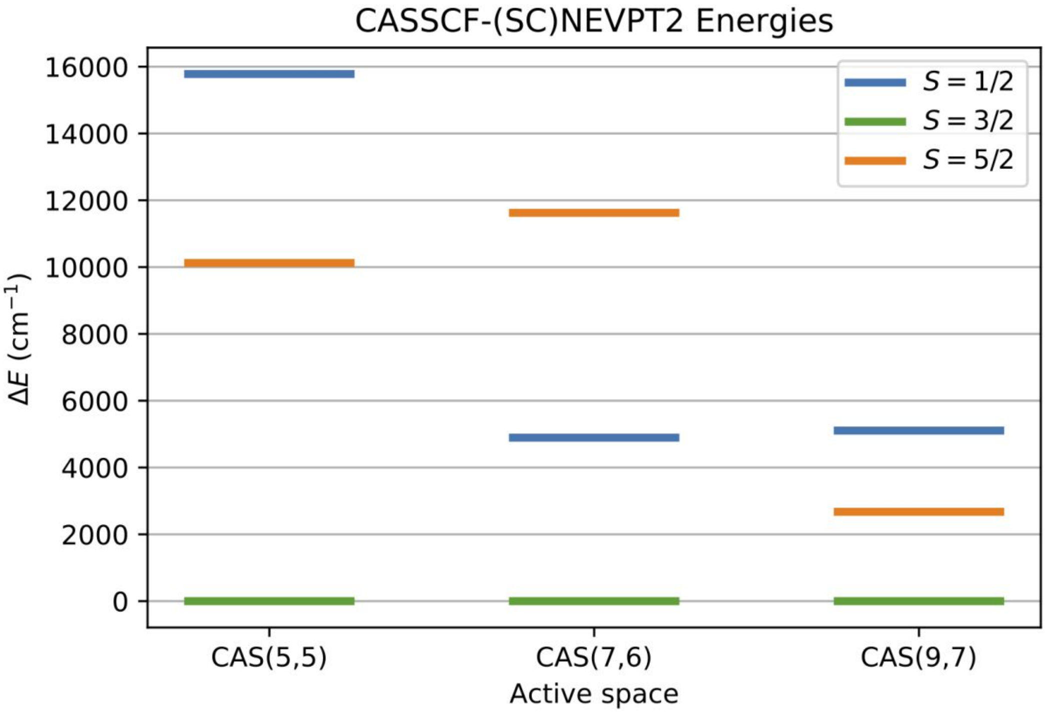

Figure S19. Relative energies of the ground doublet, quartet, and sextet states from SA-CASSCF/NEVPT2 as a function of the active space composition.

Orbital Picture of 4' from CASSCF

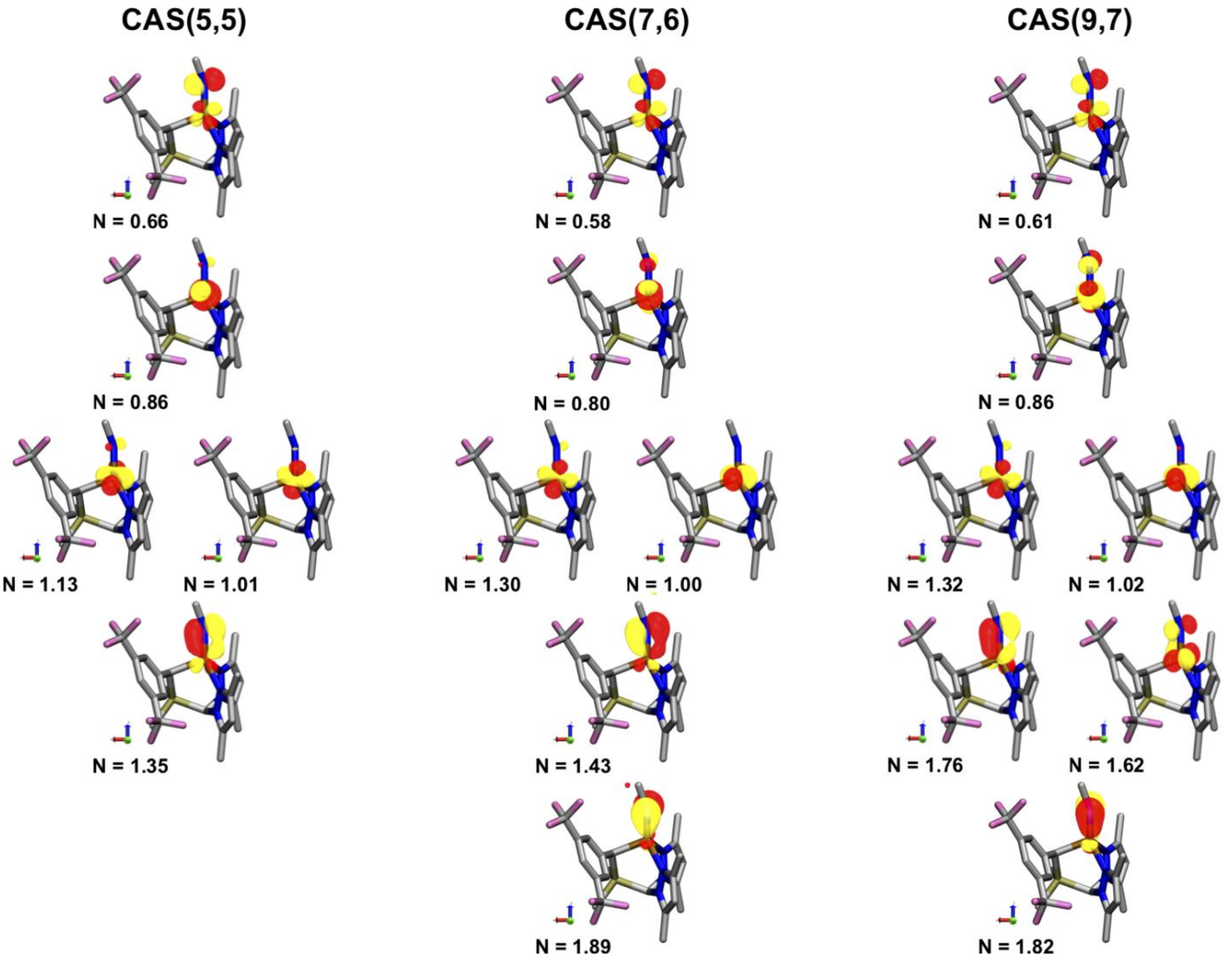

Figure S20. Active space natural orbitals from SA-CASSCF calculations of the ground doublet, quartet, and sextet states of **4'**. Natural orbitals occupation numbers averaged over all roots are presented; all isosurfaces rendered with an isovalue of 0.075 a.u..

Table S03: Dominant configurations for the ground doublet, quartet, and sextet states of **4'** from 3-root SA-CASSCF as a function of active space. Orbital labels refer to Figure S20.

S	CAS	Weight	Occupation						
			$3d_{xy}$	$3d_{yz} + 2p_y$	$3d_{xz} + 2p_x$	$3d_{z^2}$	$3d_{x^2-y^2}$	$3d_{yz} - 2p_y$	$3d_{xz} - 2p_x$
1/2	(5,5)	0.396	–	–	2	2	1	0	0
		0.198	–	–	2	1	1	1	0
		0.079	–	–	2	0	1	2	0
	(7,6)	0.640	–	2	2	2	1	0	0
		0.131	–	1	2	2	1	1	0
		0.086	–	1	1	2	1	1	1
	(9,7)	0.700	2	2	2	2	1	0	0
		0.048	2	1	1	2	1	1	1
		0.030	2	0	2	2	1	2	0
3/2	(5,5)	0.585	–	–	2	1	1	1	0
		0.147	–	–	0	1	1	1	2
		0.135	–	–	1	1	1	1	1
	(7,6)	0.600	–	2	2	1	1	1	0
		0.229	–	2	1	1	1	1	1
		0.109	–	2	0	1	1	1	2
	(9,7)	0.606	2	2	2	1	1	1	0
		0.098	2	2	1	1	1	1	1
		0.068	1	2	2	1	1	1	1
5/2	(5,5)	1	–	–	1	1	1	1	1
	(7,6)	0.975	–	2	1	1	1	1	1
		0.024	–	1	1	1	1	2	1
	(9,7)	0.684	1	2	2	1	1	1	1
		0.082	1	1	1	1	1	2	2
		0.076	2	2	1	1	1	1	1

Table S04: Löwdin spin populations for the quartet ground state of **4'**

	0% HF	10% HF	25% HF	CAS(7,6)
Fe spin pop.	2.68	2.94	3.28	3.20
N_{imido} spin pop. (p_x)	−0.12	−0.26	−0.45	−0.32
N_{imido} spin pop. (p_y)	0.19	0.14	0.07	0.04

Excited State Calculations of 4’

In order to calculate the D- and g-tensors of **4’**, we have to consider the effects of spin-orbit and, to a lesser degree, spin-spin coupling, on the ground state, which, in turn, necessitates consideration of the manifold of excited doublet and sextet states. We thus explored the convergence of the D- and g-tensors computed from SA-CASSCF/NEVPT2 calculations using the CAS(9,7) space as a function of the states included in the state interaction. Figure S21 shows the energies of the states calculated at the SA-CASSCF/NEVPT2 level as a function of the state space, while Table S05 shows the computed D- and g-tensors for each space. We also include a comparison of the experimental and calculated UV-vis-NIR spectra from the calculation including 10 doublet, 15 quartet, and 10 sextet states in Figure S22.

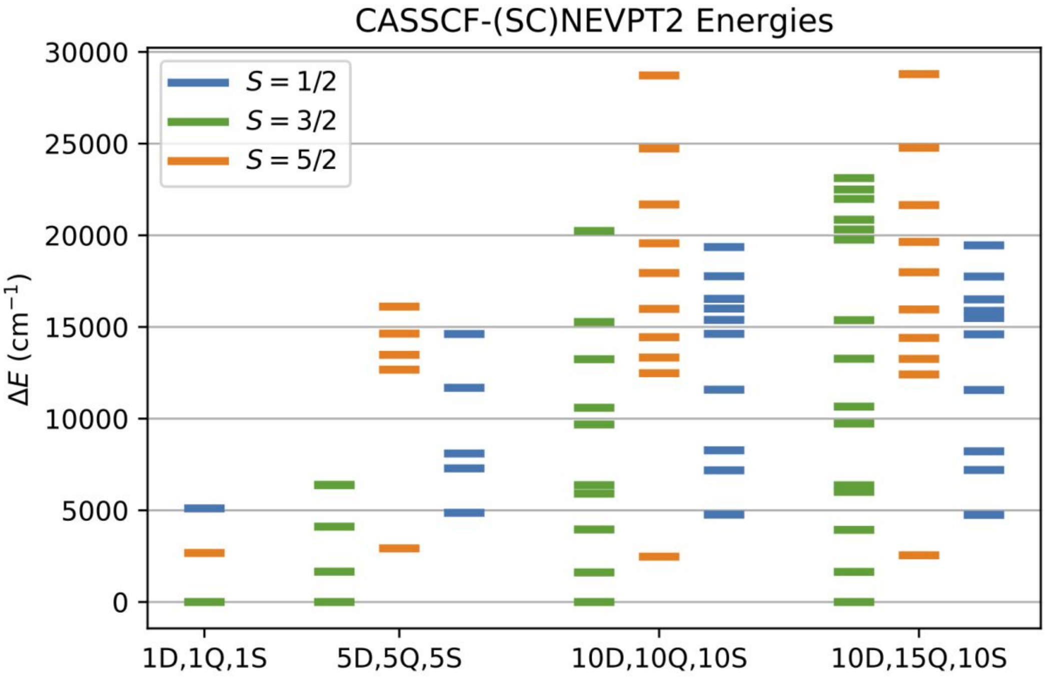

Figure S21. Spectrum of doublet (D), quartet (Q), and sextet (S) spin states as a function of the state space composition from SA-CASSCF/NEVPT2 using the CAS(9,7) active space.

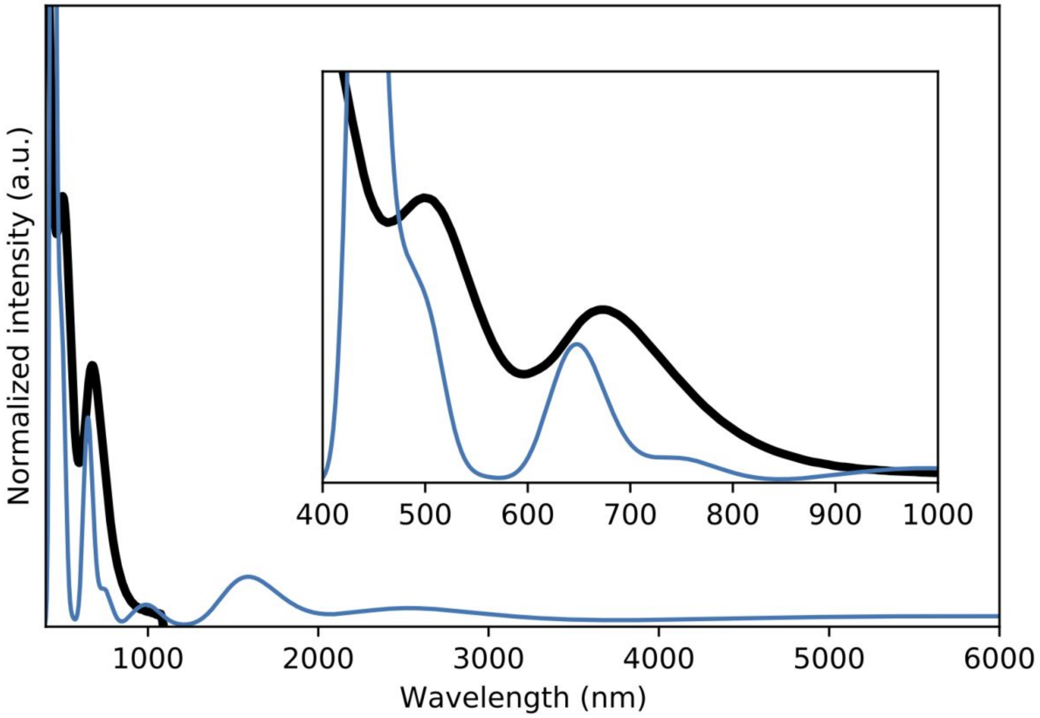

Figure S22. Comparison of the experimental UV-vis spectrum of **4** (thick black line) with the UV-vis-NIR spectrum calculated for **4’** (blue) from SA-CASSCF/NEVPT2 including the effects of spin-orbit coupling (each transition has been convoluted with a Gaussian line shape of width 1500 cm^{-1}).

Table S05: Calculated ZFS parameters and g-tensors as a function of state space composition

Number of states	D (cm^{-1})	E/D	g-factors
5D, 5Q, 5S	−32.37	0.09	1.98, 2.01, 2.42
10D, 10Q, 10S	−30.95	0.14	1.99, 2.03, 2.43
10D, 15Q, 10S	−30.05	0.15	1.99, 2.03, 2.42

We find convergence in the calculated values of D and the g-tensor already by including the first 5 quartet states in the state interaction. This appears to be due to the dominant effect of the first quartet excited state, lying only ~1600 cm^{-1} above the quartet ground state. Convergence of the ZFS rhombicity, E/D, however, requires inclusion of at least the first 6 doublet excited states. A state-specific decomposition of the computed values of D and E is provided in Table S06. Note that the sextet states only contribute indirectly to the ZFS parameters through their effect on the state energies. From this decomposition, it appears that a balanced treatment of the EPR properties of **4’** requires consideration of only the excited states lying within ca. 20,000 cm^{-1} of the ground state.

Table S06: State-specific decomposition of the ZFS parameters as a function of state space composition.[a]

S	Root	5D, 5Q, 5S		10D, 10Q, 10S		10D, 15Q, 10S	
		D (cm^{-1})	E (cm^{-1})	D (cm^{-1})	E (cm^{-1})	D (cm^{-1})	E (cm^{-1})
3/2	1	**−30.08**	0.02	**−29.83**	−0.03	**−29.23**	−0.03
	2	0.06	−0.01	0.14	−0.03	0.13	−0.02
	3	0.06	0.02	0.20	0.03	0.18	0.04
	4	0.30	0.09	0.08	−0.05	0.13	−0.03
	5	–	–	0.02	**−0.73**	0.01	**−0.72**
	6	–	–	0.00	−0.06	0.00	−0.06
	7	–	–	−0.02	0.03	−0.02	0.03
	8	–	–	0.23	0.02	0.23	0.03
	9	–	–	0.00	0.00	0.00	0.00
	10	–	–	–	–	0.16	−0.04
	11	–	–	–	–	0.02	−0.02
	12	–	–	–	–	0.03	−0.03
	13	–	–	–	–	0.04	−0.03
	14	–	–	–	–	0.00	0.00
1/2	0	−0.26	**−1.10**	−0.47	**−1.24**	−0.40	**−1.19**
	1	−0.63	**−0.67**	−0.51	**−0.53**	−0.50	**−0.52**
	2	−0.86	**−0.94**	−0.80	**−0.83**	−0.83	**−0.87**
	3	0.21	−0.03	0.19	−0.03	0.19	−0.03
	4	−0.02	0.00	−0.04	−0.03	−0.07	−0.04
	5	–	–	−0.49	**−0.52**	−0.54	**−0.65**
	6	–	–	0.34	−0.22	0.38	−0.12
	7	–	–	0.16	0.13	0.13	0.15
	8	–	–	0.97	−0.06	1.03	−0.06
	9	–	–	0.06	−0.01	0.04	−0.01

[a]Highlighted values contribute at least 10% to the total computed value of |D| or |E|.

Evaluating Metal-Ligand Covalency for 2’, 3’, and 4’

Given the good agreement between CASSCF and the 25% HF calculation for **4’** (see discussion in the main text), we have analyzed the covalency between the Fe center and the heteroscorpionate ligand in **2’**, **3’**, and **4’** *via* population analyses based upon the 25% HF DFT wavefunctions. We stress the choice of 25% HF here is strictly empirical. Table S07 presents the collected Mayer bond orders for the complexes. To complement this analysis, Table S08 collects the reduced Löwdin population analysis for the localized σ-bonding orbitals for the Fe–C and Fe–pyrazole interactions (averaged over the α and β manifolds). We observe that there is an excellent correlation (r^2 = 0.98) between the Mayer bond orders for a particular atom pair and the %Fe character in the σ-symmetry LMO (Figure S23).

Table S07: Mayer bond orders for the metal–heteroscorpionate interactions.

Bond	2’	3’a	4’
Fe–C	0.58	0.51	0.50
Fe–N_{pz1}[b]	0.30	0.23	0.16
Fe–N_{pz2}[c]	0.32	0.26	0.31

[a]All bond orders averaged over the quasi-symmetry-equivalent interactions in **3’**.
[b]N_{pz1} belongs to the longer Fe–pyrazole interaction.
[c]N_{pz2} belongs to the shorter Fe–pyrazole interaction.

Table S08: Reduced Löwdin population analysis of localized σ-bonding orbitals.

	**σ(Fe–C)**[a]		**σ(Fe–N_{pz1}[b])**		**σ(Fe–N_{pz2}[c])**	
	**%Fe**	**%L**	**%Fe**	**%N**	**%Fe**	**%N**
**2’**	18.5	71.0	15.1	70.6	15.2	70.3
**3’**[d]	16.9	72.1	13.8	71.1	14.2	70.7
**4’**	17.5	69.2	13.2	71.8	15.3	70.0

[a]The σ(Fe–C) LMO is found to be delocalized over the Si and *ipso*-C atom of the aryl substituent, so these contributions are also summed in %L (typically 7–8% of the composition can be attributed to these atoms).
[b]N_{pz1} belongs to the longer Fe–pyrazole interaction.
[c]N_{pz2} belongs to the shorter Fe–pyrazole interaction.
[d]All population analysis are averaged over the quasi-symmetry-equivalent orbitals in **3’**.

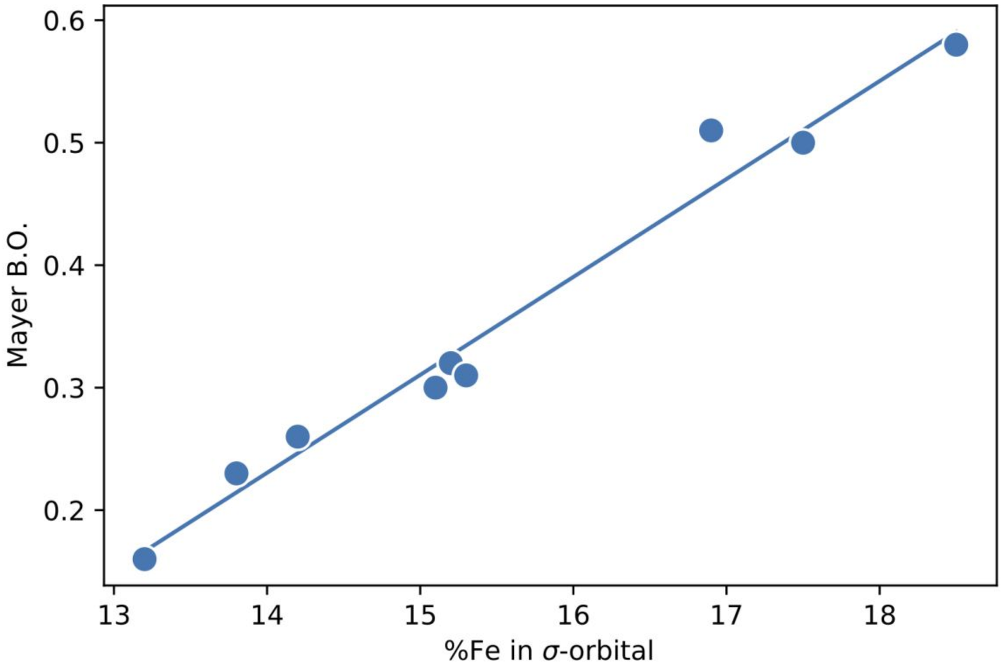

Figure S23. Correlation between Mayer bond orders and %Fe character in σ-symmetry LMOs.

References

(1) Neese, F., Prediction of Molecular Properties and Molecular Spectroscopy with Density Functional Theory: From Fundamental Theory to Exchange-Coupling. *Coord. Chem. Rev.* **2009**, *253* (5-6), 526-563.

(2) Stoian, S. A.; Vela, J.; Smith, J. M.; Sadique, A. R.; Holland, P. L.; Munck, E.; Bominaar, E. L., Mossbauer and Computational Study of an N_2-Bridged Diiron Diketiminate Complex: Parallel Alignment of the Iron Spins by Direct Antiferromagnetic Exchange with Activated Dinitrogen. *J. Am. Chem. Soc.* **2006**, *128* (31), 10181-10192.

(3) Soda, T.; Kitagawa, Y.; Onishi, T.; Takano, Y.; Shigeta, Y.; Nagao, H.; Yoshioka, Y.; Yamaguchi, K., *Ab Initio* Computations of Effective Exchange Integrals for H-H, H-He-H and Mn_2O_2 complex: Comparison of Broken-Symmetry Approaches. *Chem. Phys. Lett.* **2000**, *319* (3-4), 223-230.

(4) Vancoillie, S.; Zhao, H. L.; Tran, V. T.; Hendrickx, M. F. A.; Pierloot, K., Multiconfigurational Second-Order Perturbation Theory Restricted Active Space (RASPT2) Studies on Mononuclear First-Row Transition-Metal Systems. *J. Chem. Theory Comput.* **2011**, *7* (12), 3961-3977.

Calculated Coordinates

2

Fe	0.00277305438271	-0.00699450279867	0.00690663774153
C	1.90163911710633	-0.00391220205878	-0.91537008269511
H	2.30924724424273	0.91592582615881	-0.46519836527844
Si	1.48591118634994	0.29723310828986	-2.68003108876457
N	-0.94067033595109	1.51068889124043	-1.11383807024215
N	-1.19319363547897	1.18874512746732	-2.43443285202714
N	-1.01039469433893	-1.43666348843330	-1.17972958416119
N	-0.95161252108738	-1.23587588866900	-2.54258130797777
Cl	0.00466900950134	-0.01174012009205	2.23985625070168
F	6.43176221305434	-2.00636041137390	1.70458589820302
F	5.02274149087198	-0.97386236727191	2.92420400018599
F	5.19047717482501	-3.10657962120480	3.05881689761482
F	2.14517141526606	-5.75140995164070	-0.32507141931552
C	-1.42251001889699	2.74626818522221	-0.92476084320654
C	2.59762426856783	-1.20154808480856	-0.44045495669393
F	3.94756463610319	-5.60521928920226	-1.32545043556355
F	2.19784412033019	-4.90114816096592	-2.24507750290080
C	-1.96199715269693	3.22273928128611	-2.12935150611430
H	-2.44486127431870	4.18237435848791	-2.28756447428635
C	-0.43127044487199	0.06129234844388	-3.00412803870400
H	-0.60593632294472	0.08971969382193	-4.08966299868055
C	-1.80008608238916	2.23801184547099	-3.07981600945015
C	-1.36423878238699	3.46041132089663	0.41453847183006
C	3.51359309958109	-1.12176403623204	0.63112055919865
H	3.69987813227040	-0.15278741724720	1.09495742628474
C	-2.26968736187039	2.26044397946857	-4.52378834425800
C	-1.29112987655341	-2.37143573687997	-3.22763754151144
C	-1.44977741159751	-2.69870615243597	-1.00916868503102
C	2.38580507086518	-2.48399908568264	-0.98245204078142
H	1.67606090564940	-2.62170022592311	-1.79742398392114
C	4.16617426762841	-2.24188310725278	1.11333032319270
C	2.36493136771797	-0.80895084058289	-3.90874797086251
H	3.44305028694891	-0.80979417235703	-3.68932462871698
H	2.23548064887322	-0.44112503168814	-4.93891156390846
H	2.03469635935586	-1.85607867324013	-3.88313504847254
C	3.06260028196214	-3.59500318862622	-0.49207515604327
C	-1.62589319458091	-3.29533465973561	-2.25028008302476
H	-1.95510425528726	-4.31284219843317	-2.43829254059272
C	-1.31015270738460	-2.69093117017738	-4.72188051348007
C	5.18639906490627	-2.08564463885959	2.20142994796356
C	1.75024446034919	2.09916346769381	-3.08662191017768
H	2.82515271818364	2.33524052452089	-3.05943376427223
H	1.25225151264651	2.75241891866323	-2.35642634242152
H	1.38453730277699	2.37058996559771	-4.08893237226886
C	3.95161702167554	-3.50126799325044	0.56724976725352
H	4.47661677083531	-4.37751374982492	0.94431005106898
C	-1.72365425275043	-3.30398643612189	0.35743090582657
C	2.82926394067034	-4.93872231961090	-1.11595238349315
H	-2.96171625371173	1.43203346255702	-4.73464369162311
H	-1.43418415664065	2.19622866987346	-5.23768741835520
H	-2.79610355326376	3.20407331775906	-4.70404990789856
H	-0.78633543393811	2.87125699896131	1.13857573994822
H	-2.37596978237729	3.60701667114564	0.81877238999698
H	-0.89958725168772	4.44883355396051	0.29561545939844
H	-1.72925771881416	-3.69496677725127	-4.84060971071621
H	-0.30034382426016	-2.68125497443648	-5.15838861281767
H	-1.93106329455787	-1.98580398820550	-5.29537189909377
H	-1.37022079041606	-4.34243128509857	0.38784632918714
H	-2.80330684001022	-3.30126575547929	0.56747826939046
H	-1.21892623843400	-2.72822444283422	1.14479287981746

3

Fe	-0.00044007247146	0.00153176603037	-0.00032713962985
C	1.96520244972947	0.00103179743525	-0.79681772243097
H	2.27231707907468	1.03854723941381	-0.58264176617170

Si	1.65017604668796	-0.16217968464883	-2.59696563779539
N	-0.97422636015887	-1.63033053926948	-1.05495328269342
N	-0.76961927310403	-1.69530395779656	-2.41702728155160
N	-0.95166230344549	1.22711241933864	-1.49273338519882
N	-1.07554450927946	0.67343261262286	-2.74809158911660
N	-0.00073246112277	0.00103057478549	1.80419392958794
N	0.03913538514958	-0.02608674085038	2.98779255450638
Fe	0.03884446447553	-0.02657905221364	4.79231798682455
C	-1.92679071940386	-0.02607710432112	5.58880207458966
H	-2.24164249776785	-1.06359463940185	5.37864813665187
Si	-1.61176431134440	0.13713438005797	7.38894998918736
N	1.01262411881591	1.60528741273730	5.84693790678058
N	0.80802366679598	1.67025667529436	7.20901812623018
N	0.99007403808355	-1.25215772187471	6.28471773109290
N	1.11394891043604	-0.69847989237790	7.54008243835545
F	2.33254578198146	-5.21077866959137	0.86456849940969
F	3.10304969750692	-5.00180755631552	-1.09413116796797
F	6.93157292569260	-1.23044578474077	1.92801038979521
F	4.42168730693292	-5.41881800915041	0.48411326898476
C	-1.52653938068750	-2.80245052312467	-0.71322564207475
C	-1.62382041561311	2.38685711032193	-1.53256887880571
C	-1.62331607369441	-3.62653948850538	-1.83982901876787
H	-2.00335623309916	-4.64292505486871	-1.87183078385714
C	-0.22684252945858	-0.48697427920364	-3.04440823175072
H	-0.28149656742759	-0.65075423546083	-4.13078680395584
C	2.79283641811714	-0.95605941011646	-0.07408834461344
C	-1.13385996870013	-2.91496217295657	-2.92103379131470
C	-2.16661496608105	2.57810115161981	-2.81125941338954
H	-2.76426394574924	3.41952895835840	-3.14773756120681
C	-1.66606327837131	3.34354672795480	-0.36511871354279
C	-1.80545952317388	1.48504021396700	-3.57346633668357
C	2.66764358553813	-2.35136398795671	-0.22773618974794
H	1.88179546021173	-2.75405828937815	-0.86761151515494
C	-1.02390127605498	-3.51633917111808	-4.32768449205317
C	3.79706785593632	-0.52797004993609	0.82738482184343
H	3.92664468717318	0.54146861318614	0.99828297476875
C	-1.94543983756508	-3.13480349892029	0.70143328676017
C	4.64189536211092	-1.42097628488267	1.45989154257170
C	4.54019003644175	-2.79603365543014	1.25596657842519
H	5.22363196781406	-3.49474469656837	1.73452188890972
C	-2.18402953942121	1.20588704866603	-5.01885955698524
C	3.53344196509346	-3.23610225235553	0.40594285236096
F	5.67935860671419	-1.39501734589146	3.55857103760366
F	5.76815179307643	0.37524397683340	2.48539891520972
C	2.00545843579139	1.45826868443777	-3.45756573263340
H	3.08092737313950	1.68568359966430	-3.40435012103276
H	1.47005361683567	2.28646218658010	-2.97052459757617
H	1.72330270377694	1.45396139734390	-4.52192016438821
C	2.52378987164060	-1.60071669175125	-3.42878232311250
H	3.58543503121178	-1.62118925303466	-3.14260457608084
H	2.47734117464826	-1.50795224961040	-4.52572639153370
H	2.10215725309341	-2.57810707821447	-3.15824534864658
C	5.73265526513203	-0.92036819277092	2.35995285691486
C	3.34862288666160	-4.70649779227014	0.18512482008734
F	-2.29414138218643	5.18573138835717	3.92742234210258
F	-3.06464528893320	4.97676027329538	5.88612201297344
F	-6.89316120045481	1.20540048983501	2.86397395439087
F	-4.38328290344187	5.39377072839059	4.30787758196758
C	1.56494377471220	2.77740323231273	5.50521648350078
C	1.66222333997677	-2.41191326942396	6.32455536493355
C	1.66172045830111	3.60149220821846	6.63181985194687
H	2.04276861628046	4.61749262101861	6.66487116462793
C	0.26525426525644	0.46192897727492	7.83639257862135
H	0.32106562081773	0.62530117571870	8.92279146060118
C	-2.75442469085826	0.93101410695222	4.86607269454313
C	1.17227169430628	2.88991687715318	7.71301812607733
C	2.20501936482497	-2.60314843595489	7.60325026186851
H	2.80614647324728	-3.44269411711732	7.93805032207361
C	1.70446103008777	-3.36858985523262	5.15710334731524
C	1.84386392636647	-1.51008749537673	8.36545718437629

C	-2.62923918694639	2.32631670700133	5.01972704283419
H	-1.84136863650519	2.72797924296845	5.65748675692287
C	1.06230419636073	3.49128302208066	9.11967096668683
C	-3.75866346875253	0.50292277035293	3.96460602614589
H	-3.88772682227198	-0.56649461363638	3.79343060994042
C	1.98384421897426	3.10975620509560	4.09055755022243
C	-4.60349097265101	1.39592901057199	3.33209930971930
C	-4.50178563821396	2.77098638032860	3.53602427760743
H	-5.18502282564423	3.46970792432229	3.05712545849802
C	2.22243395058653	-1.23093432733201	9.81085040205988
C	-3.49503022764736	3.21105694798237	4.38604149995688
F	-5.64094687294258	1.36997205023857	1.23341331375183
F	-5.72974006260977	-0.40028927181013	2.30658543283632
C	-1.96705403608039	-1.48331596343699	8.24955658777500
H	-3.04272400068281	-1.70949564681453	8.19515549590555
H	-1.43302248610463	-2.31264205573856	7.76291666066781
H	-1.68618151006977	-1.47980499256960	9.31426936685934
C	-2.48538476235901	1.57567555077993	8.22076045718010
H	-3.54629045472154	1.59645927536144	7.93193475922878
H	-2.44137101144227	1.48359576419475	9.31785193659545
H	-2.06253366492165	2.55235160565129	7.94989039441199
C	-5.69424353742466	0.89532289755873	2.43203149434565
C	-3.31021848313237	4.68145050945797	4.60686602583835
H	-2.76373286945490	2.05566359682382	-5.39560432313949
H	-2.80254179775275	0.30023792154391	-5.10815707043725
H	-1.30317511867437	1.08102222800667	-5.66703168396189
H	-1.46473192391454	4.36749026961762	-0.70741858967801
H	-0.91615992813085	3.06076987699330	0.38512229643619
H	-2.65037645676319	3.33654481029147	0.12520555853521
H	2.83543015878692	-0.32186669558321	9.90014621308676
H	2.80687286668813	-2.07791275016865	10.18644381458010
H	1.34043963945600	-1.11162000789191	10.45805057488097
H	1.53790051633915	4.47657732289561	9.09382146033422
H	1.56870823049784	2.88059291073781	9.88283000241177
H	0.01400075607589	3.61473047292691	9.42918312065270
H	1.78960767719883	2.26057358045862	3.42366383164112
H	3.05604914179799	3.34912905558201	4.05435337948059
H	1.42803614374336	3.98154385991008	3.71757153015209
H	-1.58282152122649	-4.13236533266844	0.98310268402885
H	-3.04178163247755	-3.13243848834925	0.79058040753867
H	-1.53960778798527	-2.39659797420662	1.40433872038176
H	-1.52860224418232	-2.90365799371639	-5.09025999910349
H	-1.50156442430424	-4.50066798881407	-4.30289314132127
H	0.02439690540940	-3.64138787224653	-4.63647186744999
H	1.26532811424500	-2.90320785787621	4.26523361084743
H	1.13915074024771	-4.28190609832481	5.39307603863273
H	2.73738726995430	-3.66096650574341	4.92475738457159

4

Fe	0.00674559108647	0.00442372554720	0.00316989781894
C	1.96534958942568	0.00094184110611	-0.75405144265159
H	2.47897591855282	-0.58044371041601	0.02621983165295
Si	1.76922503308023	-1.05428450641056	-2.25246757954070
N	-0.88021436064664	-1.87030973139618	-0.81813107260422
N	-0.93110622317492	-1.91377538507571	-2.19895122350103
N	-0.86566155893652	0.91528843907237	-1.65637652835549
N	-0.63658862352039	0.38604350314284	-2.91122868068417
N	0.01450531802658	0.00418405619573	1.71986234284757
C	0.47930292718039	0.35361293120867	3.02802356781087
H	-0.39399987670395	0.53691857993982	3.68503868149968
H	1.09596007152761	1.27624683939862	3.05261389853256
H	1.06177450115100	-0.46266552218375	3.50264719273553
F	3.03831905144345	5.62414870293054	-3.03304961045108
F	1.20069786169486	4.58787818367986	-3.34611122587512
F	2.96492564704595	3.85804900561967	-4.24407682374310
C	2.50253908498079	4.40795710190976	-3.11501846137883
C	2.77016976866960	3.55308851727655	-1.91376525353848
C	3.54188399629213	4.04089158462275	-0.87327474967055

H	3.94002792321852	5.05319017521822	-0.89361544297771
C	3.79377006676777	3.18756856145286	0.20027853619989
C	3.30404661054747	1.89305985607037	0.21545156642341
H	3.51771324654092	1.24891965072732	1.07047327929837
C	2.49759328016484	1.37921000720709	-0.82306552698514
C	2.24022360363727	2.26361224611251	-1.89005961823485
H	1.60995061169687	1.94316487264868	-2.72050347124412
C	2.04960040643515	-2.86174268792534	-1.85296315675388
H	3.11692897656989	-3.03700617370599	-1.64810134581994
H	1.76659036361936	-3.52097813571090	-2.68757330239147
H	1.47998787836338	-3.16708869122079	-0.96570022488012
C	2.85146198772987	-0.58041018231721	-3.71741823937686
H	2.66592272545489	0.43042334954003	-4.10492257552084
H	2.73256436912890	-1.29100233855327	-4.55074263765239
H	3.90724284715669	-0.61073121987862	-3.40712402652989
C	-0.06820118045292	-0.97348592885310	-2.92764782930215
H	-0.06879586214090	-1.29477783652152	-3.97938384788480
C	-1.79763435196343	3.03825982031148	-0.69457769301137
C	-1.41172952572150	2.12953456910063	-1.85127071805926
C	-1.50772812807298	2.37741039727671	-3.22075619416387
H	-1.87688184000783	3.28282570292732	-3.69188123490218
C	-1.00179704542915	1.27446144966538	-3.89080388645278
C	-0.85894036920928	1.20463022464168	-5.41443494639734
C	-1.67499959489837	-3.34083625982401	1.06494540262466
C	-1.51108843130590	-2.98075333367957	-0.39710017482869
C	-1.94335692279935	-3.73422844193112	-1.50676281175524
H	-2.48882946497611	-4.67294894580599	-1.48129118424708
C	-1.56184069390654	-3.05413638807496	-2.63752392299674
C	-1.81668246788609	-3.44097155731612	-4.08025076768388
C	4.53899271209864	3.69507212589387	1.45374370969543
F	5.37050195389680	4.66072359308727	1.14718687112008
F	3.69936789909751	4.18836780967595	2.37036715926365
F	5.05972443191980	2.78456039035330	2.12400966832802
H	-1.19669435051714	-4.30835503288176	1.27482519875866
H	-2.73888880972598	-3.42568801237037	1.32933511885225
H	-1.21289298858883	-2.56629072694905	1.69209001385481
H	-2.39421376134357	-2.67156527149892	-4.61455116270527
H	-2.39400152868187	-4.37220528228330	-4.09520996488798
H	-0.88461163429149	-3.61341756274343	-4.64072298512558
H	-1.30484894934817	4.01432193441979	-0.79729375054799
H	-1.49313059282612	2.58162184152052	0.25697900078443
H	-2.88452827606645	3.20247997555309	-0.67762177093422
H	-1.34029877606988	2.09295894942334	-5.83515433956354
H	-1.33903241415124	0.31158203172047	-5.84200956967247
H	0.19707234916137	1.20068631932909	-5.72135996261758
